# Gut Microbiota Combined with Metabolomics Reveal the Mechanisms of Sika Deer Antler Protein on Cisplatin-Induced Hepatorenal Injury in Mice

**DOI:** 10.3390/molecules28186463

**Published:** 2023-09-06

**Authors:** Lulu Wang, Lei Li, Zhenyi Wang, Pu Zhang, Jing Zhang

**Affiliations:** 1 College of Chinese Medicinal Materials, Jilin Agricultural University, Changchun 130118, China; 20191188@mails.jlau.edu.cn (L.W.); lilei76718@126.com (L.L.); zhenyiw0324@163.com (Z.W.); zhangpu0124@163.com (P.Z.); 2 School of Medicine, Changchun Sci-Tech University, Changchun 130600, China

**Keywords:** sika deer antler protein, metabolomics, gut microbiota, cisplatin-induced liver and kidney injury

## Abstract

Cisplatin is a widely used antineoplastic drug, though its adverse effects, particularly its hepatorenal toxicity, limit its long-term application. Sika deer antler is a valuable traditional Chinese medicine (TCM) documented to possess the capacity for tonifying the kidney and regulating the liver, of which the sika deer antler protein is an important active ingredient. In this study, two protein fractions, SVPr1 and SVPr2, of sika deer antler were purified and administered to mice treated with cisplatin, and serum metabolome and fecal microbiota were measured using ultrahigh-performance liquid chromatography tandem mass spectrometry (UHPLC-MS/MS) and 16S rRNA gene sequencing. SVPr1 and SVPr2 significantly ameliorated cisplatin-induced liver and kidney injury and reduced mitochondrial dysfunction, oxidative stress, inflammatory response, and apoptosis. In addition, SVPr1 and SVPr2 impacted the gut microbiota structure of mice, significantly increasing the relative abundances of *Lactobacillus*, which deserves to be scrutinized. Moreover, SVPr1 and SVPr2 antagonism of cisplatin-induced hepatorenal injury may be related to the regulation of lysine degradation, tryptophan metabolism, and riboflavin metabolism pathways, significantly altering the levels of L-saccharopine, L-lysine, L-kynurenine, 3-methylindole, xanthurenic acid, riboflavin, and D-ribulose-5-phosphate. A correlation between the differential metabolites and *Lactobacillus* was identified. These findings increased the knowledge of the gut microbiota–metabolites axis mediated by SVPr1 and SVPr2, and may be able to contribute to the development of new therapeutic strategies for the simultaneous prevention and treatment of liver and kidney injury from cisplatin treatment.

## 1. Introduction

Cisplatin, or cis-diamminedichloroplatinum (II) (CDDP), has been employed for cancer treatment for more than 40 years as one of the most widely used drugs and is still the first choice of chemotherapeutic agents for various solid tumors, including head and neck, lung, ovarian, bladder, and testicular cancers [1]. Its antineoplastic property comes from its ability to crosslink with the purine bases on DNA and interfere with fundamental cellular processes such as replication and transcription in numerous cell types, ultimately leading to apoptosis [2]. Cisplatin also causes mitochondrial malfunction as a result of mitochondrial DNA (mtDNA) addition, causing reactive oxygen species (ROS) overproduction and boosting cytochrome c release, exacerbating oxidative stress, inflammation, and apoptosis and leading to hepatotoxicity and nephrotoxicity as severe adverse effects [3,4,5]. Cisplatin accumulates in the liver and kidney over an extended period; hence, there is a high incidence of cisplatin-induced hepatorenal impairment in clinical practice [6]. Though numerous studies have been conducted aiming to alleviate these restrictions and damage in patients, no satisfactory remedies have been described. Acute kidney injury (AKI) is often a dose-limiting adverse effect; however, liver and kidney functions interact with each other, and cisplatin hepatotoxicity has also been reported to aggravate nephrotoxicity [7,8]. Therefore, focusing on the simultaneous prevention and treatment of cisplatin hepatorenal injury may be useful for the identification of toxicity-attenuating remedies and conducive to circumventing the clinical dose limitations of cisplatin.

Deer antler is the young horn of the unossified and dense hairs of the sika deer *Cervus nippon* Temminck or *Cervus elaphus* Linnaeus and is a valuable traditional Chinese medicine (TCM) [9,10]. Chinese medicine points out that the kidney stores the essence, and the liver stores blood; deer antler enters the liver and kidney meridian, which can benefit the essence and blood, so it has the effect of tonifying the kidney and regulating the liver [9,10]. Previous research confirmed that deer antler exerted a number of properties, such as anti-stress, anti-inflammatory, and anti-aging effects, and was capable of protecting the liver and the kidney [11,12]. Modern pharmacology studies have revealed that the active ingredients in sika deer antler include proteins, peptides, amino acids, phospholipids, fatty acids, polyamines, and polysaccharides [13]. It has been reported that sika deer antler extract can protect against cisplatin-induced liver and kidney injury [14,15]. Among the variety of components comprising sika deer antler, proteins are recognized as the main effective substances. Previous research has demonstrated that protein concentrates of sika deer antler had antagonistic effects on drug-induced renal damage in vitro and in vivo [12]. These protein concentrates can antagonize acetaminophen (APAP)-induced kidney cell apoptosis, oxidative stress, and inflammation by regulating the phosphoinositide 3-kinase (PI3K)/protein kinase B (AKT), nuclear factor κΒ (NF-κΒ), and nuclear factor erythroid 2-related factor 2 (Nrf2) signaling pathways [12]. Since side effects and resistance remain the two inherent challenges of cisplatin administration, combination therapies are being explored to improve the drug’s effectiveness against cancer. Therefore, the synergistic antagonism of cisplatin hepatorenal toxicity by sika deer antler active proteins has become a point of interest.

The gut microbiota is a complex physiological ecosystem that affects the health of the host and has become a new biomarker [16,17]. Moreover, the gut microbiota is considered to be an important metabolic organ of the human body, and the specific microbial metabolites secreted by bacteria, as well as their final downstream products, can impact the host’s phenotype and physiology [18,19]. In addition, the combination of metabolomics and gut microbiome analysis is an effective strategy to explore the potential mechanisms of drug effects in TCM [20]. It has become increasingly recognized that in addition to the myriad effects of cisplatin, it can destabilize host gut microbial communities, leading to noticeable perturbations in metabolic levels; however, certain drugs can relieve cisplatin-induced liver and kidney damage by modulating the gut microbiota–metabolites axis [21,22,23]. However, it remains unclear what roles the sika deer antler protein plays in repairing cisplatin-induced liver and kidney damage; furthermore, how gut bacteria and serum metabolites are affected by sika deer antler proteins in these processes has not yet been reported. Thus, it is necessary to characterize the potential mechanisms whereby sika deer antler proteins antagonize the liver and kidney injury resulting from cisplatin treatment through combined metabolomics and gut microbiota analyses.

In this study, we isolated and obtained the sika deer antler protein fractions SVPr1 and SVPr2 and elucidated their protective effects against the hepatic and renal injury induced by cisplatin treatment. Additionally, we applied an integrated approach consisting of untargeted serum metabolomics analysis combined with 16S rRNA gene sequencing of the gut microbiota to explore the relationship between intestinal microbiota and metabolites for the first time in this model of hepatorenal injury. From the perspective of the gut microbiota–metabolites axis, the potential mechanisms whereby SVPr1 and SVPr2 are capable of blocking cisplatin-induced mitochondrial dysfunction, inflammation, and oxidative stress in the liver and kidney were revealed. With the feasibility and safety of fecal microbiota transplantation (FMT) established, interruption of the amplification of cascade reactions by targeting the gut microbiome is a potential novel strategy to compensate for the side effects from chemotherapy drugs. Therefore, understanding the characteristics of the gut microbiota and its derived metabolites affected by sika deer antler protein may provide valuable insights that will contribute to the development of this TCM as well as to the improvement of cisplatin therapy.

## 2. Results

### 2.1. Isolation and Molecular Weight Identification of Sika Deer Antler Proteins

**Figure 1:**
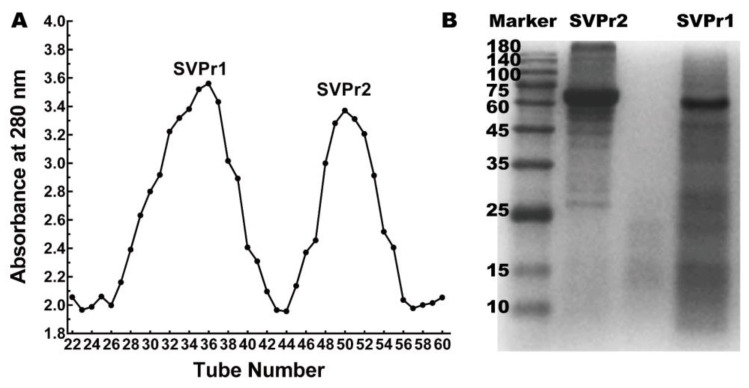
Sika deer antler protein separation and molecular weight identification. (**A**) Sephadex G-100 column chromatographic elution curve of the total protein of sika deer antler showing two fractions, SVPr1 and SVPr2. (**B**) Sodium dodecyl sulfate–polyacrylamide gel electrophoresis (SDS-PAGE) image of SVPr1 and SVPr2.

Column chromatography was employed to separate the sika deer antler proteins into two protein fractions, SVPr1 and SVPr2 (Figure 1A). The molecular weights of SVPr1 and SVPr2 were evaluated by sodium dodecyl sulfate–polyacrylamide gel electrophoresis (SDS-PAGE), which revealed their principal compositions concentrated in the molecular weight range of 10–180 kDa, with variations in their ranges of protein molecular weights. However, there were also similarities between them; for example, SVPr1 and SVPr2 were both rich in proteins in the molecular weight ranges of 60–75 kDa (Figure 1B).

### 2.2. Protective Effects of SVPr1 and SVPr2 on Cisplatin-Induced Liver and Kidney Injury

**Figure 2:**
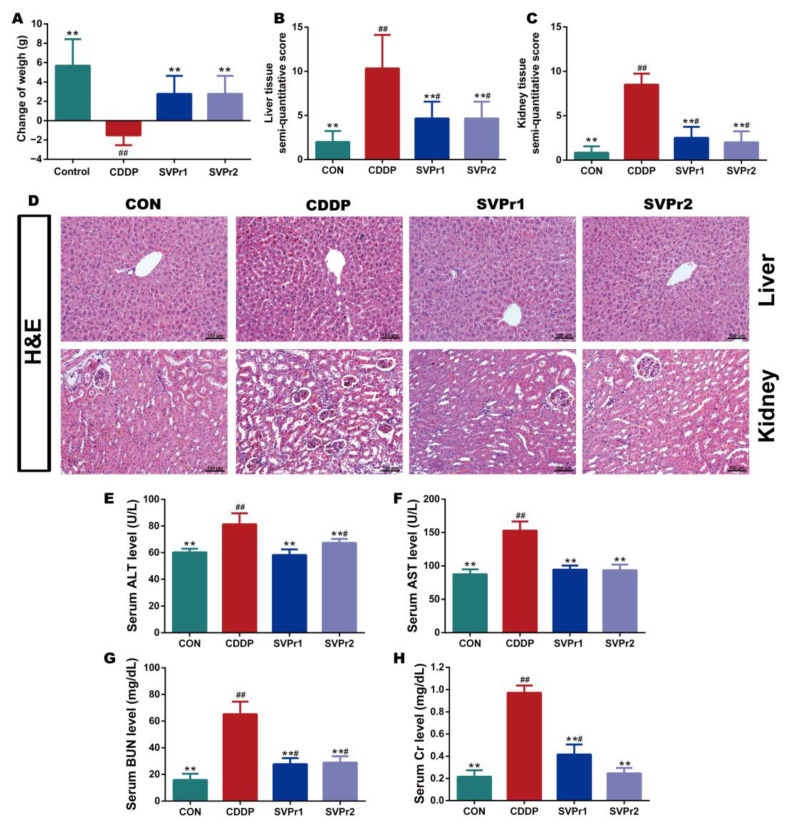
Protective effects of SVPr1 and SVPr2 on cisplatin-induced liver and kidney injury in mice. (**A**–**H**) The control (CON), cisplatin (CDDP), and SVPr1 and SVPr2 treatment groups were compared in terms of changes in body weight (**A**), liver tissue injury score (**B**), kidney tissue injury score (**C**), hematoxylin and eosin (H&E) staining of liver and kidney tissues (**D**), serum concentrations of liver and kidney injury markers, including alanine transaminase (ALT) (**E**), aspartate aminotransferase (AST) (**F**), blood urea nitrogen (BUN) (**G**), and creatinine (Cr) (**H**). Data are presented as mean ± standard deviation (SD) (*n* = 6 per group). ^#^: *p* < 0.05 as compared to the CON group; ^##^: *p* < 0.01 as compared to the CON group; **: *p* < 0.01 as compared to the CDDP group.

Based on the pharmacological model of cisplatin-induced liver and kidney injury in mice, the protective effects of SVPr1 and SVPr2 on liver and kidney injury resulting from cisplatin were determined. Cisplatin administration led to weight loss in mice, whereas treatments with SVPr1 and SVPr2 slowed the trend. Ultimately, the weights of mice in the CDDP group were significantly lower than their initial weights, while the weights of mice in the SVPr1 and SVPr2 groups were significantly higher than their initial weights (Figure 2A, *p* < 0.01). Mice treated with cisplatin exhibited an increase in liver index, whereas no change in kidney index was found, and the liver index was significantly reduced by treatment with SVPr1 and SVPr2 (Table S1, *p* < 0.01). Moreover, hematoxylin and eosin (H&E) staining revealed that the liver and kidney tissue structures of the control (CON) group were organized, while the liver cord tissue of the CDDP group was damaged, with obvious evidence of liver water-like degeneration, hepatocyte necrosis, and inflammatory infiltration. Additionally, the kidney tissue of the CDDP-group mice showed damaged renal tubules, brush margin loss, glomerular integrity destruction, apoptosis, and inflammatory infiltration. In contrast, administration of SVPr1 and SVPr2 significantly restored the histopathological changes induced by cisplatin treatment and preserved the liver and kidney tissue integrity of mice (Figure 2B–D, *p* < 0.01). Furthermore, the serum concentrations of the liver and kidney injury markers aspartate aminotransferase (AST), alanine transaminase (ALT), blood urea nitrogen (BUN), and creatinine (Cr) of mice in the CDDP group were significantly higher compared to those in the CON group, while administration of SVPr1 and SVPr2 decreased the concentrations of these injury markers (Figure 2E–H, *p* < 0.01).

### 2.3. Effects of SVPr1 and SVPr2 on Mitochondrial Dysfunction, Oxidative Stress, Inflammation, and Apoptosis in Cisplatin-Induced Liver and Kidney Injury

**Figure 3:**
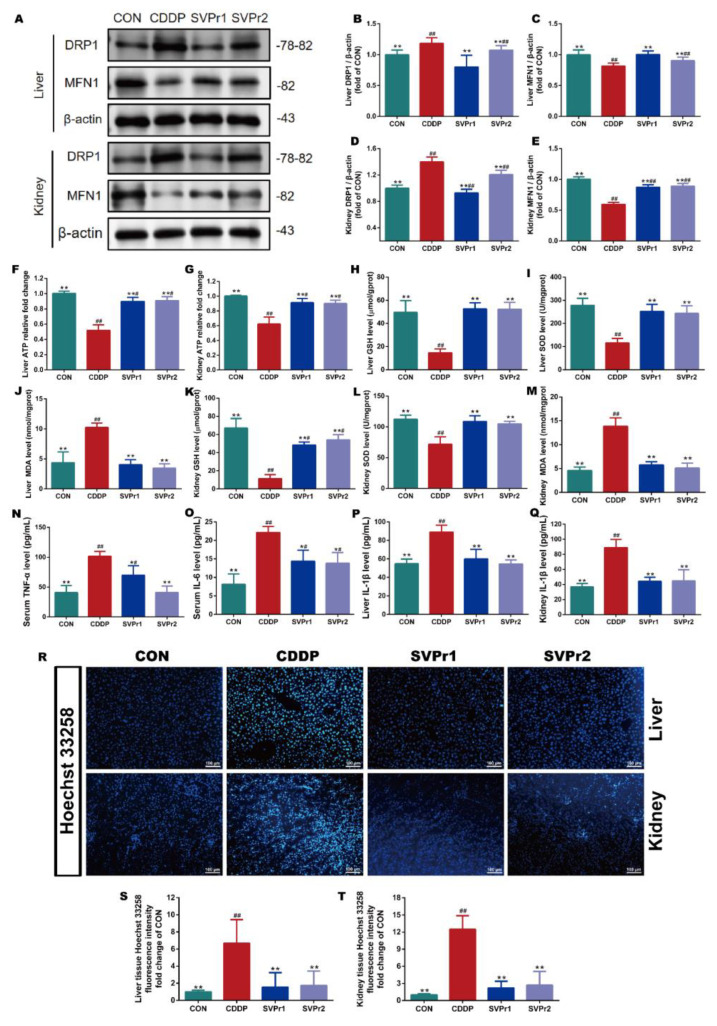
Effects of SVPr1 and SVPr2 on cisplatin-induced mitochondrial dysfunction, oxidative stress, inflammation, and apoptosis in the liver and kidney. (**A**–**E**) The CON, CDDP, and SVPr1 and SVPr2 treatment groups were compared in terms of DRP1 and MFN1 protein expression in the liver and kidney. (**F**,**G**) The CON, CDDP, and SVPr1 and SVPr2 treatment groups were compared in terms of ATP contents in the liver (**F**) and kidney (**G**). (**H**–**M**) The CON, CDDP, and SVPr1 and SVPr2 treatment groups were compared in terms of oxidative stress, including liver glutathione (GSH) (**H**), liver superoxide dismutase (SOD) (**I**), liver malondialdehyde (MDA) (**J**), kidney GSH (**K**), kidney SOD (**L**), and kidney MDA (**M**). (**N**–**Q**) The CON, CDDP, and SVPr1 and SVPr2 treatment groups were compared for inflammation, including serum TNF-α (**N**), serum IL-6 (**O**), liver IL-1β (**P**), and kidney IL-1β (**Q**). (**R**–**T**) The CON, CDDP, and SVPr1 and SVPr2 treatment groups were compared for apoptosis using Hoechst 33258 fluorescent staining (**R**) in hepatic (**S**) and renal (**T**) tissues. Data are presented as mean ± SD (*n* = 6 per group). ^#^: *p* < 0.05 as compared to the CON group; ^##^: *p* < 0.01 as compared to the CON group; *: *p* < 0.05 as compared to the CDDP group; and **: *p* < 0.01 as compared to the CDDP group.

The mechanisms of cisplatin-induced hepatorenal toxicity are related to mitochondrial dysfunction, oxidative stress, and inflammation [4,5]. Therefore, we evaluated the roles of SVPr1 and SVPr2 in improving mitochondrial dysfunction, oxidative stress, and inflammation in the liver and kidney induced by cisplatin. Dynamin-related protein 1 (DRP1) and mitofusin-1 (MFN1) protein expression levels, as well as ATP content, were utilized to evaluate mitochondrial function. Compared with the CON group, the ATP contents in the liver and kidney tissues of mice in the CDDP group were significantly reduced but were markedly increased following SVPr1 or SVPr2 administration, indicating that SVPr1 and SVPr2 effectively inhibited the ATP depletion due to cisplatin-induced impairment of mitochondrial function (Figure 3F,G, *p* < 0.01). In addition, cisplatin induced a significantly increased DRP1 and decreased MFN1 expression, whereas SVPr1 and SVPr2 pretreatment dramatically restored mitochondrial DRP1 and MFN1 protein expression levels, indicating that SVPr1 and SVPr2 had the ability to reverse cisplatin interference in dynamic mitochondrial homeostasis (Figure 3A–E, *p* < 0.01). These results demonstrated that SVPr1 and SVPr2 could protect the function of mitochondria in hepatocytes and kidney cells, antagonizing cisplatin’s hepatorenal toxicity. Moreover, the concentrations of glutathione (GSH) and superoxide dismutase (SOD) in the liver and kidney tissues of cisplatin-induced mice decreased, while the concentrations of malondialdehyde (MDA) increased (Figure 3H–M, *p* < 0.01). After SVPr1 and SVPr2 administration, the concentrations of GSH and SOD increased significantly, while the concentrations of MDA decreased significantly, suggesting that treatment reduced the level of cisplatin-induced oxidative stress in the liver and kidney and improved the antioxidative defense capacity of mice (Figure 3H–M, *p* < 0.01). Furthermore, evaluation of inflammatory markers revealed that the levels of tumor necrosis factor α (TNF-α) and interleukin 6 (IL-6) in serum and interleukin 1β (IL-1β) in the liver and kidney tissues were increased in the mice of the CDDP group, while these elevations were significantly reversed by SVPr1 or SVPr2 administration (Figure 3N–Q, *p* < 0.05 or *p* < 0.01), demonstrating that treatment reduced the occurrence of cisplatin-induced liver and kidney inflammatory response events. In addition, Hoechst 33258 fluorescent staining revealed that CDDP treatment aggravated apoptosis in mouse hepatic and renal tissues, an effect that was alleviated by SVPr1 or SVPr2 administration (Figure 3R–T, *p* < 0.01).

### 2.4. Effects of SVPr1 and SVPr2 on the Gut Microbiota of Mice with Cisplatin-Induced Liver and Kidney Injury

Next, 16S rRNA gene sequencing was performed to evaluate the effects of SVPr1 and SVPr2 on the gut microbiota of mice with cisplatin-induced liver and kidney injury, and returned 1,514,030 reads. Under the condition of 99% similarity, the reads were clustered into operational taxonomic units (OTUs) of classified species, with a total of 4613 OTUs obtained (Figure S1A). The Chao 1, observed features, and Shannon indices were positively correlated with the species richness of the sample gut microbiota. The gut microbiota α diversity increased after cisplatin administration, though with no statistical difference compared to the CON group (Figure S1B–D). However, based on principal coordinates analysis (PCoA) and non-metric multidimensional scaling (NMDS) analysis, the similarity of gut microbiota (β diversity) assessment identified significant separation of microbial communities between the CON, CDDP, SVPr1, and SVPr2 groups, revealing marked differences in the composition of bacterial communities among the four groups (Figure S1E,F). The closer the distance, the higher the sample similarity, and the farther the distance, the lower the sample similarity. The relatively long distances between the CDDP and CON groups indicate that cisplatin induced considerable changes in the intestinal microbiota structure of the mice, while the relatively close distances between the SVPr1 and SVPr2 administration groups and the CON group indicate that both protein fractions may be capable of regulating gut microbiota to antagonize cisplatin toxicity (Figure S1E,F).

**Figure 4:**
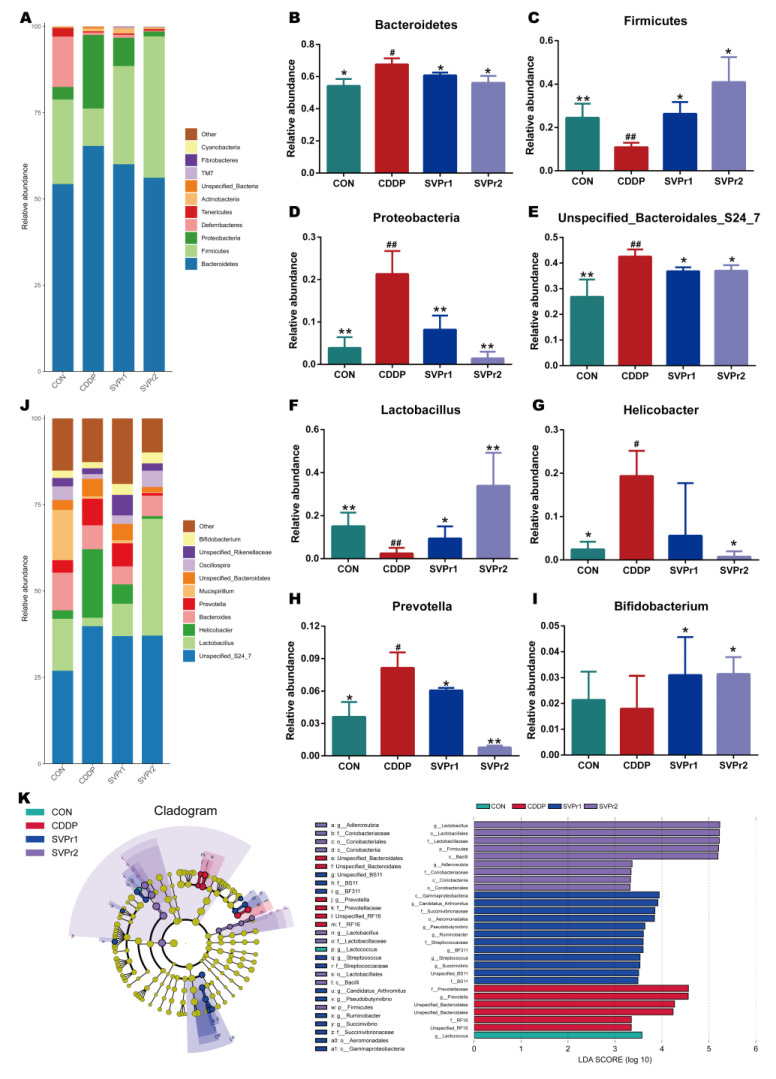
Compositional alteration of the gut microbiota comparing the control, cisplatin, SVPr1, and SVPr2 groups. (**A**) Histogram of relative species abundance at the phylum level (top 10). (**B**–**D**) Differential gut microbiota at the phylum levels. (**E**–**I**) Differential gut microbiota at the genus levels. (**J**) Histogram of relative species abundance at the genus level (top 10). (**K**) Cladogram plotted from linear discriminant analysis effect size (LEfSe) analysis showing the phylogenetic distribution (**left**) and LDA score diagram (**right**). LDA scores (log10) ≥ 2 and *p* < 0.05 are listed. In the cladogram plot, colored circles and shadings indicate the significantly enriched bacterial taxa obtained in each corresponding group. The size of each point indicates the negative logarithm (base 10) of the *p*-value, with bigger point size suggesting more significance (lower *p*-value). Data are presented as mean ± SD. ^#^: *p* < 0.05 as compared to the CON group; ^##^: *p* < 0.01 as compared to the CON group; *: *p* < 0.05 as compared to the CDDP group; and **: *p* < 0.01 as compared to the CDDP group.

Moreover, the changes in gut microbiota structure were compared between each group. At the phylum level, the species abundance of gut microbiota in mice altered after cisplatin administration. We analyzed the high-abundance gut microbiota and found that following cisplatin administration, the relative abundances of Bacteroidetes and Proteobacteria were significantly upregulated, whereas the relative abundance of Firmicutes was significantly downregulated. Compared with the CDDP group, administration of SVPr1 and SVPr2 significantly downregulated Bacteroidetes and Proteobacteria and upregulated the relative abundance of Firmicutes (Figure 4A–D, *p* < 0.05 or *p* < 0.01). The relative abundances of Firmicutes and Bacteroidetes were more than 75% in each sample, and were the most dominant phyla among the intestinal bacteria of mice. Previous studies have shown that an imbalance of the ratio between Firmicutes and Bacteroidetes can affect energy metabolism, which may be one of the reasons for the weight loss that occurs in mice treated with cisplatin [24]. In addition, the pharmacological model of kidney yang deficiency syndrome featured changes in the gut microbiota, with a decrease in Firmicutes and an increase in Bacteroidetes, consistent with the traditional therapeutic efficacy of sika deer antler on tonifying kidney yang [25]. Consistently, Proteobacteria is among the most abundant phyla, and previous reports have indicated that the abundance of Proteobacteria increased with cisplatin-induced intestinal damage [26]. Furthermore, at the genus level, following cisplatin administration, the relative abundances of *Unspecified_Bacteroidales_S24_7*, *Helicobacter*, and *Prevotella* were significantly increased and that of *Lactobacillus* was significantly decreased compared to the control group. In contrast, following administration of SVPr1 and SVPr2, the relative abundances of *Unspecified_Bacteroidales_S24_7* and *Prevotella* decreased significantly, while that of *Lactobacillus* increased significantly (Figure 4E–J, *p* < 0.05 or *p* < 0.01). Additionally, compared to the CDDP group, SVPr2 administration significantly reduced the abundance of *Helicobacter*, while SVPr1 administration also contributed to a reduction in *Helicobacter* abundance, though the difference was not significant (Figure 4G, *p* < 0.05). Interestingly, cisplatin reduced the abundance of *Bifidobacterium* compared to the CON group, but without a significant difference, whereas SVPr1 and SVPr2 treatments significantly increased the abundance of *Bifidobacterium* (Figure 4I, *p* < 0.05). These results parallel the previous findings that cisplatin induced the apoptosis of *Lactobacillus*, which may be the main reason for the significant decrease in intestinal *Lactobacillus* in mice observed after cisplatin administration [27].

Linear discriminant analysis (LDA) and LDA effect size (LefSe) analysis further revealed the characteristic gut microbiota in the different subgroups (Figure 4K). In the different subgroups, higher LDA values indicated greater differences, and taxa with LDA ≥ 2 were considered significantly different. Only one of the characteristic gut microbiota in the CON group, *Lactococcus*, belonged to the Firmicutes. In the cisplatin group, six characteristic gut microbiota were identified, all of which belonged to the Bacteroidetes, with *Prevotella* and *Unspecified_Bacteroidales* as the main characteristic gut microbiota. The SVPr1 dosing group included 12 species of Firmicutes, Proteobacteria, and Bacteroidetes, with *Gammaproteobacteria* as the main characteristic organism. In the SVPr2 dosing group, a total of nine species were distributed in the Firmicutes and Actinobacteria, among which the main characteristic bacteria was *Lactobacillus*. The variability of the characteristic gut microbiota between groups suggests that the improvement of cisplatin-induced liver and kidney injury following SVPr1 and SVPr2 administration may be related to their increased abundance of Firmicutes and decreased abundance of Bacteroidetes at the phylum level and their increased abundance of *Lactobacillus* at the genus level (Figure 4K).

**Figure 5:**
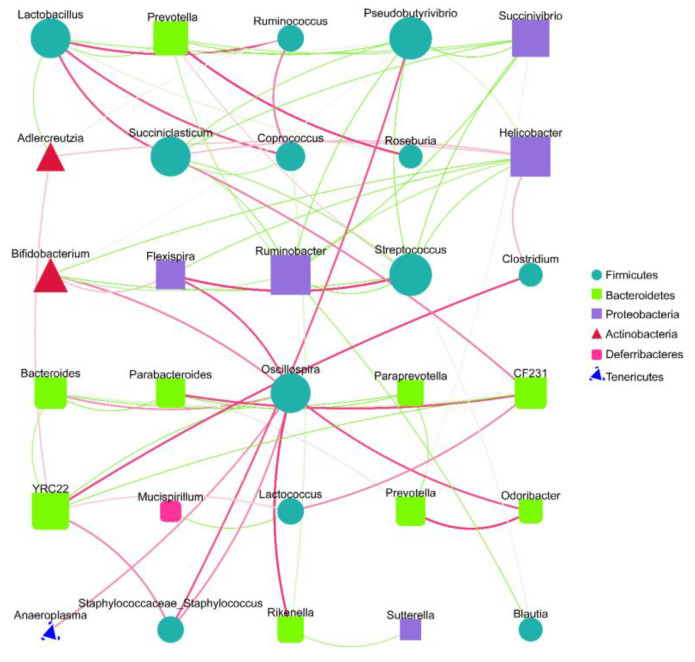
Species interaction network diagram at the genus level (top 30). Different nodes represent different genera; the node size represents the average relative abundance of the genus, and the nodes within a given phylum have the same colors. There is a positive relationship between the thickness of the line and the correlation coefficient of the specie’s interaction. The green lines represent negative correlations between two genera, while red lines represent positive correlations.

Spearman analysis based on the relative abundance at the genus level and the microbiota interaction network maps that were constructed revealed that there were extensive interactions between the sample gut microbiota (Figure 5). The nodes of the Firmicute and Bacteroidete groups were higher in number in the interactive network constructed from the top 30 genera of relevance, indicating that both phyla contributed substantially to the inter-collaborative network. In addition, there were significant negative correlations between *Lactobacillus* and *Prevotella* and between *Helicobacter* and *Bifidobacterium*, suggesting that there may be certain inhibitory relationships between the two, and their changes in abundance may have opposite trends influenced by environmental factors, consistent with our observed experimental results. *Lactobacillus*, *Prevotella*, *Helicobacter*, and *Bifidobacterium*, which were previously screened as high-abundance inter-group differential bacteria, were all determined to be involved in the interaction network, with degree values of 8, 6, 8, and 6, respectively, which were relatively high, indicating that they played crucial roles in the interaction network (Figure 5). These results suggest that SVPr1 and SVPr2 could exert important impacts on the structure of cisplatin-induced gut microbiota in mice by regulating the abundances of the above four bacteria.

**Figure 6:**
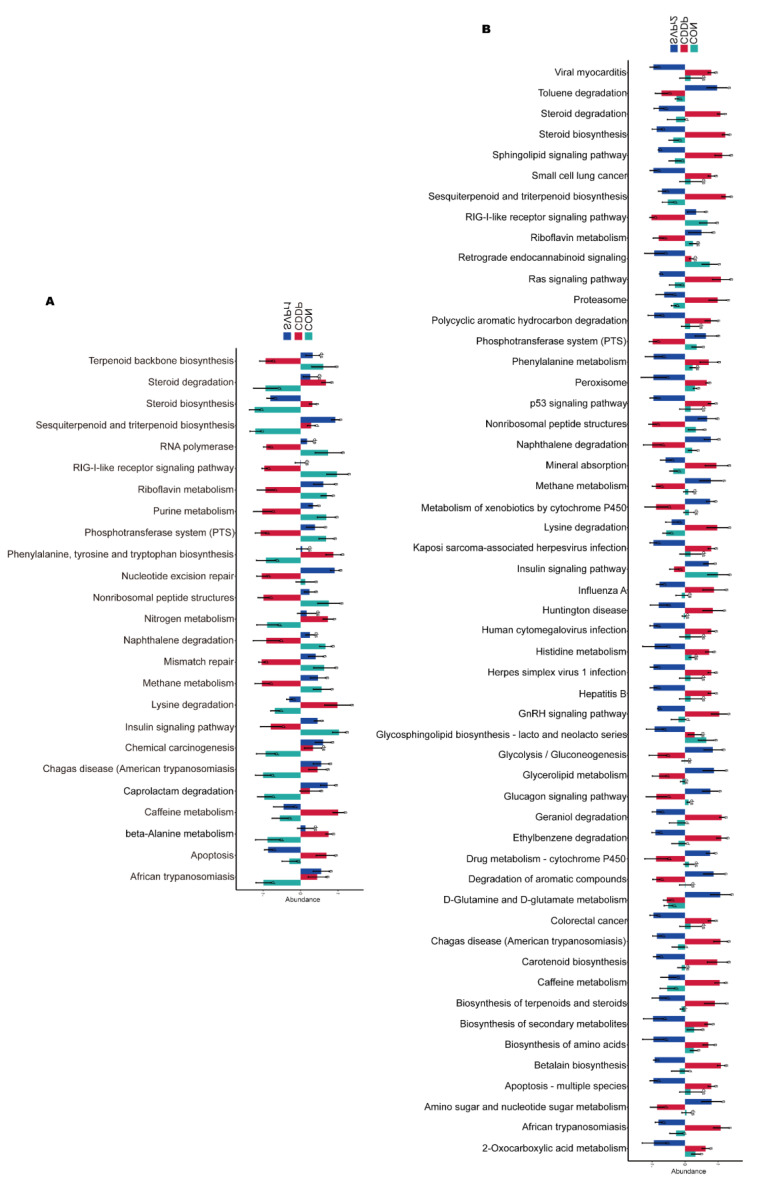
Functional enrichment analysis of gut microbiota based on phylogenetic investigation of communities by reconstruction of unobserved states (PICRUSt). (**A**) Comparison of different functions between the CON, CDDP, and SVPr1 groups. (**B**) Comparison of different functions between the CON, CDDP, and SVPr2 groups.

Phylogenetic investigation of communities by reconstruction of unobserved states (PICRUSt)-based analysis was performed to predict the functions of the gut microbiota and to compare the differential functions between groups (Figure 6A,B). There were significant differences in 25 pathways among the CON, CDDP, and SVPr1 comparison groups and in 53 pathways among the CON, CDDP, and SVPr2 comparison groups. Interestingly, there were differences in both apoptosis and insulin signaling pathways in the comparisons of the two groups, with SVPr1 and SVPr2 administration promoting the downregulation of the abundances of genes related to the apoptosis pathway and the upregulation of genes related to the insulin signaling pathway compared to the CDDP group. The insulin signaling pathway involves the PI3K/AKT cascade responses [28]. Previous studies have indicated that sika deer antler proteins can attenuate pharmacogenetic kidney cell apoptosis by regulating the PI3K/AKT signaling pathway [12]. Based on the results of the above functional difference analysis, it can be speculated that these effects may be related to their regulation of the gut microbiota. In connection with the subsequent metabolomics analysis, we focused on the differences in the regulation of metabolic pathways between groups. A total of 15 metabolic pathways were significantly different among the CON, CDDP, and SVPr1 administration groups, among which 13 metabolic pathways showed significant recovery trends after SVPr1 administration (Figure 6A). In addition, a total of 29 metabolic pathways were significantly different among the CON, CDDP, and SVPr2 administration groups, of which 23 metabolic pathways showed recovery trends after SVPr2 administration (Figure 6B). Intriguingly, the same differential functional metabolic pathways between the two comparison groups included lysine degradation, riboflavin metabolism, and steroid biosynthesis. Compared with the CDDP group, SVPr1 and SVPr2 administration promoted the downregulation of lysine degradation and steroid biosynthesis gene abundance, whereas genes related to riboflavin metabolism were upregulated. These results indicate that following cisplatin-induced liver and kidney injury, the structure of the murine gut microbiota changed significantly, while SVPr1 and SVPr2 treatment could antagonize cisplatin hepatic and renal toxicity by modulating or improving gut microbiota homeostasis.

### 2.5. Effects of SVPr1 and SVPr2 on the Metabolites in Mice with Cisplatin-Induced Liver and Kidney Injury

We then performed mass spectrometry to assess whether deer antler proteins disrupted the metabolome in mice treated with cisplatin. The total number of ion flow diagrams for ultrahigh-performance liquid chromatography tandem mass spectrometry (UHPLC-MS/MS) in positive and negative ion modes are shown in Figure S2. To ensure the stability and reliability of the UHPLC-MS/MS system and data, the quality control-based random forest signal correction (QC-RFSC) algorithm of the statTarget package in R was used to correct the signal peaks of the features (i.e., each metabolite) of each sample during the analysis, and the correction effect of each metabolite was recorded. The samples were well aggregated in the principal component analysis (PCA) score plot, with quality control (QC) samples close to the center of the axis and no signal drift, and the signal intensity remained constant, indicating that the system was in a stable state from the first to the last sample, confirming that the data could be used for subsequent studies (Figure S3).

**Figure 7:**
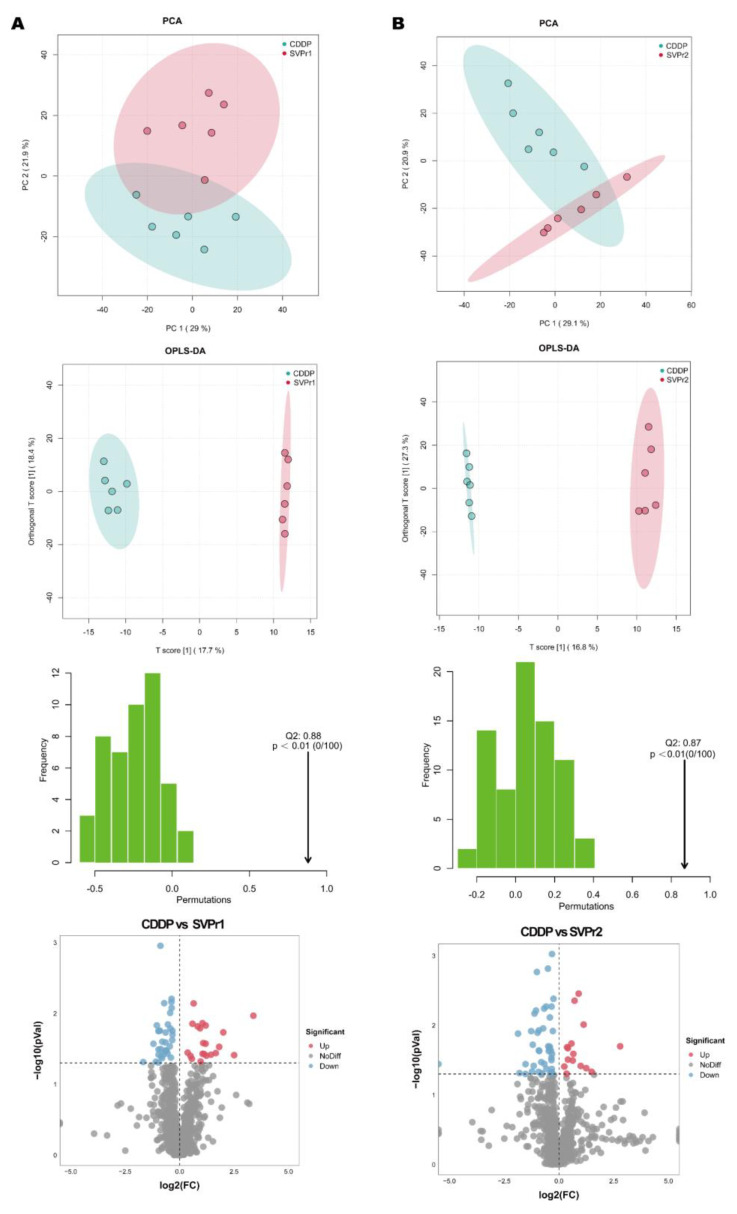
Effects of SVPr1 and SVPr2 on the metabolites in mice with cisplatin-induced liver and kidney injury. (**A**,**B**) Principal component analysis (PCA), orthogonal partial least squares discriminant analysis (OPLS-DA), OPLS-DA permutation test results, and volcano plots of the SVPr1 (**A**) and SVPr2 (**B**) groups compared to the CDDP group, respectively. Each symbol in the score plots refers to a sample (n = 6 per group), and the samples are color-coded according to their group information. In the volcano plots, the red dots represent significantly upregulated metabolites (OPLS-DA VIP > 1 and *p* < 0.05), the blue dots represent significantly downregulated metabolites (OPLS-DA VIP > 1 and *p* < 0.05), and the gray dots represent insignificantly changed metabolites (*p* > 0.05).

Differential metabolite screening was first executed by multivariate analysis. The PCA score plots revealed that the point clouds of the samples from the SVPr1 and SVPr2 administration groups were clearly distributed in different areas than those from the CDDP group, indicating that the drug interventions altered the metabolic profiles of the mice and that the metabolite composition structures of the two subgroups differed significantly (Figure 7A,B). Orthogonal projections to latent structures discriminant analysis (OPLS-DA), a supervised identification method, was used to further screen the differential metabolites and revealed that the sika deer antler protein treatment groups and the CDDP model samples were well separated from each other, indicating that SVPr1 and SVPr2 administration interfered with the metabolite types and quantities induced by cisplatin treatment in mice (Figure 7A,B). Additionally, in the permutation tests of OPLS-DA, the Q2 of the SVPr1 and CDDP comparison group was 0.88, and that of the SVPr2 and CDDP comparison group was 0.87, both greater than 0.5 and approaching 1, and the *p*-values were less than 0.01, indicating that the model had good discrimination effectiveness and could meet the prediction capacity of the data matrix (Figure 7A,B). Volcano plots constructed from the results of the univariate statistical analysis revealed the variation tendencies of the differential metabolites in the comparison groups (Figure 7A,B). Based on the threshold of OPLS-DA variable importance in the projection (VIP) value > 1 and *p*-value < 0.05, 48 and 49 differential metabolites were screened in the SVPr1 and SVPr2 administration groups, respectively, compared with the CDDP group (Tables S2 and S3).

**Figure 8:**
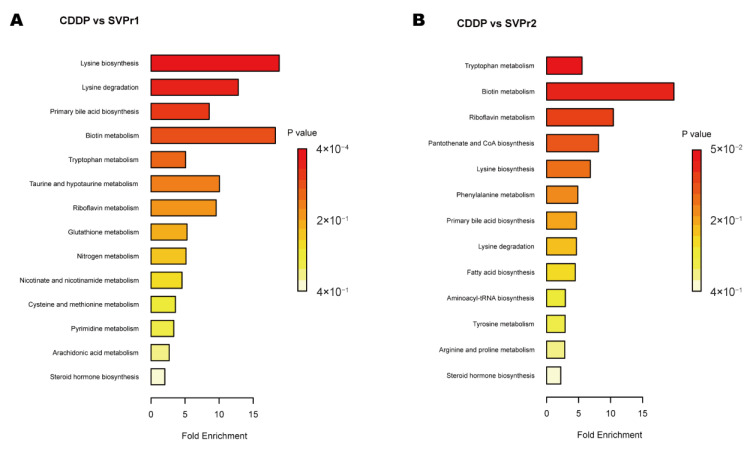
Enriched KEGG pathways of differential metabolites in cisplatin-treated mice administered SVP. (**A**) SVPr1 group compared to the CDDP group. (**B**) SVPr2 group compared to the CDDP group.

Kyoto Encyclopedia of Genes and Genomes (KEGG) enrichment analysis showed certain similarities in the types of metabolic pathways enriched in the differential metabolites in the two comparison groups, suggesting some commonality in the metabolic mechanisms of SVPr1 and SVPr2 antagonism of cisplatin-induced hepatorenal toxicity, which may be related to the similarity in their composition of bioactive substances (Figure 8). The main metabolic pathways involved amino acid metabolism, energy metabolism, lipid metabolism, and cofactor and vitamin metabolism. The differential metabolites between the SVPr1 and CDDP groups were associated with 14 metabolic pathways, and the differential metabolites between the SVPr2 and CDDP groups were associated with 13 metabolic pathways, suggesting that these metabolic pathways may be key pathways in the prevention and treatment of cisplatin-induced liver and kidney injury by SVPr1 and SVPr2. Notably, both SVPr1 and SVPr2 could mitigate cisplatin-induced hepatic and renal injury by regulating tryptophan metabolism. Compared with the CDDP group, after SVPr1 administration, the levels of the tryptophan metabolism-related metabolites L-kynurenine and 3-methylindole were decreased, while that of xanthurenic acid was increased, and after SVPr2 administration, the levels of L-kynurenine and 3-methylindole were decreased, while the levels of L-tryptophan and xanthurenic acid were increased (Table 1 and Table 2). Interestingly, compared with the results of the PICRUSt analysis of gut microbiota, both the differential metabolites and differential functions of the intestinal microbiota were associated with lysine degradation and riboflavin metabolism (Figure 6 and Figure 8). Compared with the cisplatin group, the serum levels of the lysine degradation products L-saccharopine and L-lysine were reduced in both the SVPr1 and SVPr2 administration groups. In addition, the serum levels of riboflavin were decreased and the levels of D-ribulose 5-phosphate, a substance that promotes riboflavin catabolism, were increased in the SVPr1 and SVPr2 administration groups (Table 1 and Table 2), suggesting that SVPr1 and SVPr2 administration affected the occurrence of lysine degradation and riboflavin metabolism, which may impact their regulation of gut microbiota-related gene composition.

### 2.6. Correlation Analysis of Gut Microbiota and Differential Metabolites in Cisplatin-Treated Mice Administered Sika Deer Antler Proteins

**Figure 9:**
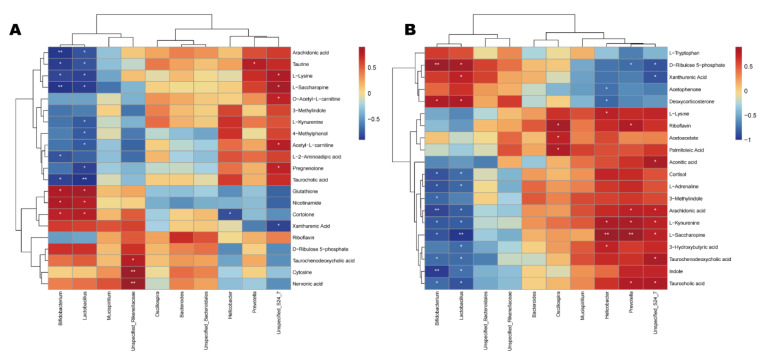
Correlation relationships between gut microbiota and differential metabolites in cisplatin-treated mice administered sika deer antler proteins. (**A**,**B**) Hierarchica- clustering heat maps of Spearman correlation analysis of gut microbiota at the genus level and differential metabolites from the SVPr1 (**A**) and SVPr2 (**B**) groups compared to the CDDP group. Rows represent the discriminative metabolites, and columns represent the dominant bacterial genera. *: *p* < 0.05; **: *p* < 0.01.

The differential metabolites of the CDDP and SVPr1 and the CDDP and SVPr2 comparison groups were subjected to Spearman correlation analysis in association with the dominant gut microbiota of each comparison group. Hierarchical-clustering heat maps of the correlation analysis revealed the covariances between the dominant gut microbiota (genus level) and the differential metabolites (Figure 9). The results showed certain correlations between the key distinguishing metabolic products and differential bacterial populations of high abundance in the two comparison groups, again suggesting that SVPr1 and SVPr2 could affect host metabolism by modulating gut microbiota to antagonize cisplatin-induced liver and kidney injury. Specifically, in the CDDP and SVPr1 comparison groups, the tryptophan metabolite xanthurenic acid was significantly negatively correlated with *Unspecified_Bacteroidales_S24_7*, and *L-kynurenine* was significantly negatively correlated with *Lactobacillus*. For the metabolites of the lysine degradation pathway, L-saccharopine and L-lysine were significantly positively correlated with *Unspecified_Bacteroidales_S24_7* and negatively correlated with *Lactobacillus* and *Bifidobacterium*. Moreover, in the CDDP and SVPr2 comparison groups, the tryptophan metabolite L-kynurenine was significantly positively correlated with *Unspecified_Bacteroidales_S24_7*, *Prevotella*, and *Helicobacter* and negatively correlated with *Lactobacillus* and *Bifidobacterium*. Additionally, 3-methylindole was significantly and negatively correlated with *Bifidobacterium*. Xanthurenic acid was negatively correlated with *Unspecified_Bacteroidales_S24_7* and positively correlated with *Lactobacillus*. Furthermore, L-lysine, a metabolite of the lysine degradation pathway, was significantly and positively correlated with *Helicobacter*. L-saccharopine was significantly and positively correlated with *Unspecified_Bacteroidales_S24_7*, *Prevotella*, and *Helicobacter* and had negative correlations with *Lactobacillus* and *Bifidobacterium*. Regarding riboflavin metabolism, riboflavin was significantly and positively correlated with *Prevotella* and *Oscillospira*. In addition, D-ribulose 5-phosphate showed significant negative correlations with *Unspecified_Bacteroidales_S24_7* and *Prevotella* and significant positive correlations with *Lactobacillus* and *Bifidobacterium* (Figure 9).

## 3. Discussion

Cisplatin induces dysregulation of the gut microbiota and associated metabolites; therefore, correcting these dysfunctions is of great significance in antagonizing the organ toxicity associated with its administration [22]. It has been reported that cisplatin-induced liver and kidney injury in mice disturbed the structure of the gut microbiota, elevating the populations of harmful bacteria and affecting the levels of metabolites, particularly those involved in antioxidant capacity and inflammation [23], consistent with our findings. We also demonstrated that treatment with SVPr1 and SVPr2 could protect liver and kidney tissues from cisplatin-related toxic injury by inhibiting mitochondrial dysfunction, oxidative stress, inflammation, and apoptosis, which correlated with their modulatory effects on the cisplatin-induced imbalance of gut microbiota and metabolites. Collectively, these findings indicate that sika deer antler protein treatment may alleviate cisplatin-induced hepatorenal injury via the regulation of the gut microbiota, significantly increasing the relative abundances of *Lactobacillus* and noteworthy, as well as in the mediation of metabolites, for particularly influencing the lysine degradation, tryptophan metabolism, and riboflavin metabolism pathways, highlighting the effects of sika deer antler proteins and providing a new strategy to prevent and treat cisplatin-induced organ injury.

Cisplatin induces an imbalance in the levels of oxidative stress and inflammatory response in the liver and kidney [4,5], whereas SVPr1 and SVPr2 administration could reduce cisplatin-induced adverse reactions by modulating the gut microbiota and metabolic pathways. To explore the possibility that the mitigation of cisplatin-induced injury by sika deer antler protein treatment may be initiated by pathogenic microbes and establish correlations between the presence of certain bacteria with the relief of hepatic and renal toxicity in mice, we performed microbiome analysis based on 16S rRNA sequencing. In the β-diversity analysis, which considered the differences in the composition of bacterial communities among the different groups, the clusterings of CDDP and sika deer antler protein-treated samples were well separated, by relatively long distances, suggesting that SVPr1 and SVPr2 treatments significantly altered the species composition and abundance of gut microbiota. *Helicobacter* and *Prevotella* are considered to be characteristic microorganisms of several inflammatory liver and kidney diseases, including hepatitis, immunoglobulin A (IgA) nephropathy, and alcoholic liver injury [29,30,31,32,33]. Additionally, *Helicobacter* infection is a major risk factor during cancer treatment, not only inducing inflammatory lesions in healthy tissues but also promoting cisplatin resistance in tumors [34]. *Lactobacillus* and *Bifidobacterium* are typically beneficial bacterial genera. *Bifidobacterium* can exert nephroprotective effects through immunomodulation and also attenuate liver damage [35,36]. Cisplatin treatment induces the apoptosis of *Lactobacillus*, which may be the main reason for the significant decrease in the *Lactobacillus* level in the intestines of mice after cisplatin administration; restoration of intestinal *Lactobacillus* abundance is thought to be a favorable approach to repair cisplatin-induced oxidative stress and inflammatory damage in the liver and kidney [23,27,37]. In support of this hypothesis, previous studies have confirmed that *Lactobacillus rhamnosus* GG extenuated deoxynivalenol-induced kidney oxidative damage in weaned piglets [38], and *Lactobacillus acidophilus* LA14, which is used worldwide, alleviated liver injury in rat and mouse models [39]. In our research, gut microbiota correlation analysis revealed significant negative correlations between *Lactobacillus* and *Prevotella* and between *Bifidobacterium* and *Helicobacter*. SVPr1 and SVPr2 treatments significantly inhibited the cisplatin-induced expansion of *Helicobacter* and *Prevotella* populations in the intestines of mice, while promoting *Lactobacillus* and *Bifidobacterium* expansion, which may be one of the mechanisms mediating their antagonism of cisplatin-induced liver and kidney inflammation and oxidative stress injury. Notably, compared to the model group, the main characterized bacteria in the other groups included *Lactobacillus*. Based on these findings, it would be interesting to explore the individual roles of certain gut microorganisms in susceptibility to cisplatin-induced hepatorenal toxicity, and to better tailor TCM for personalized medicine.

The liver and kidney are the major organs of drug metabolism and require large numbers of mitochondria to maintain their energy requirements. Increasing numbers of scholars believe that mitochondrial damage is a key pathological mechanism of cisplatin-induced liver and kidney injury; therefore, new therapeutic strategies targeting mitochondrial protection are urgently needed [40,41]. Lysine is an essential amino acid in humans that is mainly catabolized through the saccharopine pathway in hepatic mitochondria [42]. The lysine degradation product L-saccharopine has been demonstrated to be toxic to mitochondria, and its adequate catabolism is essential for mitochondrial homeostasis [43]. Previous reports indicated that cisplatin toxicity was related to its interference with amino acid metabolism, and changes in the metabolic levels of L-lysine and L-saccharopine were detected, implicating the lysine degradation pathway [21,44,45]. Beier et al. [46] showed that L-saccharopine was increased during ischemia-reperfusion kidney injury (IRI) and suggested that excessive L-saccharopine accumulation may be a key mechanism of mitochondrial dysfunction in IRI. Notably, the cisplatin-induced increase in serum L-saccharopine accumulation in mice may also contribute to its induction of liver and kidney injury, particularly mitochondrial dysfunction. Moreover, a previous study showed that reducing L-saccharopine accumulation had a protective effect on hypertensive nephropathy [47]. In addition, the probiotic bacteria *Lactobacillus* can protect the host from the noxious products of lysine degradation by identifying oxidized amino acids as harmful species and responding by upregulating stress pathways [48]. Indeed, *Lactobacillus* supplementation in patients with tuberculosis has been shown to reduce L-saccharopine levels and modulate inflammatory cytokines [49]. Consistently, the gut microbiota and metabolomics analyses in our study revealed that SVPr1 and SVPr2 antagonism of cisplatin-induced hepatorenal toxicity was associated with the lysine degradation pathway, with downregulation of associated gut microbiota gene abundances and levels of the related products L-saccharopine and L-lysine. Meanwhile, SVPr1 and SVPr2 administration increased the abundance of *Lactobacillus* in the intestines of mice, and L-saccharopine and L-lysine levels were negatively correlated with *Lactobacillus* abundance. Therefore, it is hypothesized that SVPr1 and SVPr2 administration may correct the impairment of host lysine degradation and reduce L-saccharopine accumulation by increasing *Lactobacillus* abundance, representing a potential mechanism of their action in antagonizing cisplatin-induced hepatic and renal mitochondrial dysfunction. These findings provide a new perspective for subsequent studies to explore novel approaches to protect mitochondria against cisplatin-induced liver and kidney injury through regulating the lysine degradation pathway.

Tryptophan is an essential amino acid that is primarily metabolized in the gastrointestinal tract. A previous report indicated that the tryptophan metabolite xanthurenic acid was dose-dependently decreased in the renal medulla after cisplatin exposure [50]. Functional annotation of KEGG pathways in the metabolomics analysis revealed that tryptophan metabolism was significantly enriched in both comparison groups, suggesting that it may represent a common key pathway by which SVPr1 and SVPr2 treatments combat cisplatin-induced hepatorenal toxicity. Tan et al. [51] profiled the complete tryptophan pathway in cisplatin-induced AKI and concluded that tryptophan metabolism was closely related to cisplatin-induced nephrotoxicity, suggesting that intervention in tryptophan metabolism represented a new potential strategy for the pharmacological treatment of cisplatin-induced kidney injury. Changes in tryptophan and its metabolites have been associated with cisplatin-induced kidney injury, regulating oxidative stress, inflammation, and the immune response [52]. Furthermore, the gut microbiota plays a role in improving disorders of tryptophan metabolism. Large numbers of bacterial species, including those among the Bacteroides, can produce indole derivatives of tryptophan, which are ligands of the aryl hydrocarbon receptor (AhR), and can consequently activate the local downstream expression of interleukin 22 (IL-22). The Lactobacillus–tryptophan–AhR axis has been reported to mediate intestinal homeostasis [53], and to affect immunoregulatory T cells [54] and the improvement of alcoholic hepatitis [55]. *Lactobacillus* supplementation has also been shown to improve intestinal inflammation induced by abnormal tryptophan metabolism [53,54]. Our results showed that, in contrast to the trend of tryptophan-related metabolism regulation by cisplatin, SVPr1 administration decreased 3-methylindole and L-kynurenine levels and increased xanthurenic acid levels, while SVPr2 administration decreased 3-methylindole and L-kynurenine levels and increased tryptophan and xanthurenic acid levels. Moreover, correlations between related differential metabolites and Lactobacillus were identified, indicating that both SVPr1 and SVPr2 could interfere with tryptophan metabolism to antagonize cisplatin-induced hepatonephritis and oxidative damage by affecting the relative abundance of Lactobacillus.

The results of the differential functional enrichment analysis of gut microbiota and differential metabolites revealed that SVPr1 and SVPr2 antagonized the hepatorenal toxicity of cisplatin treatment and were related to riboflavin metabolism, demonstrating that SVPr1 and SVPr2 antagonism of cisplatin-induced hepatorenal toxicity was associated with riboflavin metabolism. Riboflavin, also known as vitamin B2, is an essential nutrient that must be obtained from the diet or from metabolites of the commensal gut microbiota [56]. Moreover, riboflavin exists in two co-enzymatic forms, flavinadenine dinucleotide (FAD) and flavinadenine mononucleotide (FMN), which are used in metabolic redox reactions by different enzyme systems. The probiotics Lactobacillus and Bifidobacterium are capable of synthesizing riboflavin [56]. In addition, Hassan et al. [57] showed that riboflavin dose-dependently ameliorated cisplatin-induced hepatic and renal toxicity under photo illumination. Previous studies have also shown that promoting riboflavin metabolism can be beneficial for maintaining redox homeostasis in mice [58]. Consistently, after SVPr1 and SVPr2 administration, the abundance of genes related to gut microbiota riboflavin metabolism increased, while the serum level of riboflavin decreased and the content of D-ribulose 5-phosphate increased, suggesting that SVPr1 and SVPr2 promoted riboflavin metabolism, which was beneficial for repairing cisplatin-induced oxidative stress injury in the liver and kidney.

Despite our findings, the present study has several limitations. First, the sample size was relatively small. Because the identification of high-quality biomarkers requires rigorous studies with adequate sample sizes, larger sample sizes should be used in future research to identify potential novel biomarkers. Second, although correlations were identified between gut dysbiosis and serum metabolites in cisplatin-induced liver and kidney injury treated with sika deer antler proteins, the causal and regulatory relationships among them have not been clarified. To better understand the causative role of specific bacteria species, antibiotics should be administrated to deplete the gut microbiota, and fecal microbiota transplantation can be performed prior to cisplatin treatment. Finally, it will be necessary to elucidate the active components and optimal dosage of sika deer antler proteins in the treatment of cisplatin-induced hepatorenal injury in combination with pharmacology. Further research on how these particular pathways are integrated and the discovery of the cell type-specific roles of the critical molecules involved in oxidative stress and inflammation will enable a comprehensive understanding of the complex mechanisms of cisplatin toxicity, potentially leading to the development of targets to protect the liver and kidney without compromising the chemotherapeutic efficacy of cisplatin.

## 4. Materials and Methods

### 4.1. Material and Reagents

Sika deer antlers were purchased from a deer farm in Shuangyang in Jilin Province, and sika deer antler proteins were produced in the laboratory. Cisplatin (purity ≥ 99%) was purchased from Sigma-Aldrich (St. Louis, MO, USA). The assay kits for Cr, BUN, SOD, GSH, and MDA were purchased from Nanjing Jiancheng Bioengineering Institute (Nanjing, China). ELISA kits to evaluate TNF-α, IL-6, and IL-1β were purchased from Meilian Biotechnology (Shanghai, China). The kits for bicinchoninic acid (BCA) protein content assay, ATP content detection, Hoechst 33258 fluorescent staining, and H&E staining were obtained from Solarbio Science & Technology (Beijing, China). The primary antibodies of DRP1 and MFN1 were purchased from Cell Signaling Technology (Danvers, MA, USA), and β-actin was purchased from Solarbio Science & Technology (Beijing, China). Goat Anti-Mouse IgG specific Antibody (HRP Conjugate) was provided by KT Life technology (Shenzhen, China). Biowest agarose was purchased from SenBeiJia Biological Technology (Nanjing, China). FastPfu polymerase was obtained from TransGen Biotech (Beijing, China). An AxyPrep DNA Gel Extraction Kit was purchased from Axygen Biosciences (Hangzhou, China). High-performance liquid chromatography (HPLC)-grade methanol, formic acid, and ammonium acetate were obtained from Thermo Fisher Scientific (Waltham, MA, USA).

### 4.2. Methods

#### 4.2.1. Extraction and Separation of Sika Deer Antler Protein

For protein separation, the Soxhlet reflux extraction method was used. The solid-to-liquid ratio was 1:10. Ethyl ether and 95% ethanol were used to degrease the samples for 3 h. Then, three reflux extractions with 35% ethanol were carried out over 2 h, and the filtrates were merged. The concentration of alcohol was adjusted to 80% (*v*/*v*), and the solution was kept at 4 °C overnight. The precipitate was centrifuged at 4500 rpm and 4 °C for 10 min. Then, the precipitate was evaporated until no alcohol remained. Total sika deer antler protein was obtained by freeze-drying. The protein was further separated and purified using a Sephadex G-100 column and eluted with distilled water at a flow rate of 0.5 mL/min. The absorbance of the effluent components was detected at 280 nm. According to the spectra, two sika deer antler protein fractions, SVPr1 and SVPr2, were isolated and obtained.

#### 4.2.2. SDS-PAGE Analysis

SDS-PAGE was performed to identify the molecular weights of SVPr1 and SVPr2. The 5 mg/mL sample solutions of all the sika deer antler proteins were prepared, and were then mixed with 30 μL non-denaturing loading buffer (*v*/*v* = 4:1), boiled at 100 °C for 10 min, kept at room temperature, and set aside to cool. The solution was then centrifuged before utilization. An SDS-PAGE system consisting of a 5% stacking gel and a 10% separating gel was used, and the voltages were 80 and 120 V, respectively, with a loading volume of 15 μL. After electrophoresis, the gel was washed 3 times with deionized water for 15 min each wash, fixed with 10 mL of fixative for 30 min, and stained with Coomassie brilliant blue R-250 at 20 rpm/min for 40 min. It was then washed with deionized water 3 times for 10 min each time. Next, the gel was immersed in a destaining solution to elute overnight. Finally, a gel imaging system was used for visualization and analysis (Tanon 5200, Shanghai Tianneng Technology, Shanghai, China).

#### 4.2.3. Animals and Procedures

All animal experiments were approved by the Animal Ethics Committee of Jilin Agricultural University and were carried out in strict accordance with the “Guidelines for the Care and Use of Laboratory Animals of Jilin University”. Eight-week-old male ICR mice (20–25 g) were purchased from Changchun Yisi Laboratory Animals (animal certificate number: SCXK (Ji) 2020-0002, Changchun, China). The animals were acclimated under standard conditions for 7 days with free access to food and water and at controlled temperature (23 ± 2 °C) and humidity (50–60%) conditions, with a 12 h light/dark cycle. The animals were divided into 4 groups (*n* = 6). The CON group was administered distilled water (0.01 mL/g) by gavage for 10 consecutive days, and an equal amount of 0.9% saline was injected intraperitoneally after 2 h of gavage administration on day 7. The CDDP group was administered distilled water (0.01 mL/g) by gavage for 10 consecutive days, and an equal amount of cisplatin (20 mg/kg) was injected intraperitoneally after 2 h of gavage administration on day 7. The SVPr1 and SVPr2 groups were administered SVPr1 and SVPr2 (60 mg/kg) by gavage for 10 consecutive days, respectively, and cisplatin (20 mg/kg) was administered intraperitoneally after 2 h of gavage administration on day 7. After administration on day 9, feces were collected from mice, with each mouse in a sterilized single metabolic cage, for 4 h. The fresh feces were immediately frozen in liquid nitrogen until DNA extraction. The body weights of the mice were recorded daily, and after the last administration on day 10, blood was drawn retro-orbitally from the mice in each group after 12 h of fasting without water. The blood was centrifuged at 3500 r/min for 10 min at 4 °C after standing for 20 min at room temperature, to collect serum samples. Liver and kidney samples were also collected for subsequent experiments.

#### 4.2.4. Determination of Liver and Kidney Injury Markers, Oxidative Stress Indicators, and Inflammatory Factors

The assays were performed following strictly the instructions of the commercially available kits. The levels of ALT, AST, Cr, and BUN in mouse serum were determined using the microplate method to evaluate the levels of liver and kidney injury. The microplate method was also used to determine the levels of SOD, GSH, and MDA content in liver and kidney tissues for the evaluation of oxidative stress levels. ELISA kits for the determination of TNF-α and IL-6 contents in serum and IL-1β contents in liver and kidney tissues were used to evaluate inflammation levels. A BCA kit was used for protein quantification.

#### 4.2.5. Histopathological Analysis

Liver and kidney tissues were placed in 10% formalin solution, fixed, and embedded in paraffin, and sections with a thickness of 5 μm were prepared (Leica RM 2235, Leica, Wetzlar, Germany). H&E staining was performed following strictly the instructions of the commercially available kit. The sections were sealed with neutral gel and photographed by observation under a microscope with 200× magnification (Leica DM 2500, Leica, Wetzlar, Germany).

#### 4.2.6. Hoechst 33258 Fluorescent Staining

Hoechst 33258 fluorescent staining was also performed to detect the extent of apoptosis. As described previously, liver and kidney tissue sections with a thickness of 5 μm were dewaxed (Leica RM 2235, Germany). Hoechst 33258 fluorescent staining was performed following strictly the manufacturer’s instructions. A Leica microscope was used to detect apoptosis (Leica, Wetzlar, Germany). Cells with dense staining and fluorescence in Hoechst 33258 dye were determined to be apoptosis-positive.

#### 4.2.7. Assays for ATP Content

The liver and kidney tissues of the mice were collected and crushed in an ice bath. The determinations of ATP content were performed using colorimetric assays, following the manufacturers’ instructions. The ATP content of tissue was calculated according to the sample mass (μmol/g).

#### 4.2.8. Western Blot Analysis

Lysing mouse liver and kidney tissues using RIPA lysis solution combined with protein phosphatase inhibitors yielded total protein samples. The total protein content in the tissues was measured using the BCA protein quantification kit. Protein samples were separated on 12% SDS-PAGE gels and electrophoretically transferred to polyvinylidene fluoride (PVDF) membranes. The PVDF membranes were then sealed with 5% skimmed milk at room temperature for at least 2 hours before being incubated overnight at 4 °C with DRP1 (1:1000), MFN1 (1:1000), and β-actin (1:1500) primary antibody solutions. The membranes were then incubated for 2 h at room temperature with the HRP-conjugated secondary antibody solution. Finally, we used Quantity One software (Bio-Rad Laboratories, Hercules, CA, USA) to quantify the signal intensities of protein bands.

#### 4.2.9. Metabolomics

A 100 μL aliquot serum sample was added with 100 μL of 80% methanol aqueous solution. After vortexing and shaking, the samples were allowed to stand in an ice bath for 5 min and centrifuged at 15,000× *g* for 15 min at 4 °C. A certain amount of supernatant was taken and diluted with mass-spectrometry-grade water to 53% of methanol, which was then centrifuged at 15,000× *g* and 4 °C for 15 min. The supernatant was collected to perform UHPLC-MS/MS analysis. Thermo Vanquish UHPLC chromatograph (Thermo Hypersil Gold column, 100 × 2.1 mm, 1.9 μm; Thermo Fisher Scientific) and Thermo QE series mass spectrometer (Thermo Fisher Scientific) were used. An equal volume of sample from each experimental sample was mixed as the QC sample. The aqueous 53% methanol solution was used instead of the experimental sample as the blank sample, and the pre-treatment procedure was the same as that of the experimental sample. A Hypersil Gold column (C_18_) was used with a column temperature of 40 °C and a flow rate of 0.2 mL/min. In positive mode, mobile phase A was 0.1% formic acid, and mobile phase B was methanol. In negative mode, mobile phase A was 5 mM ammonium acetate, pH 9.0, and mobile phase B was methanol. The elution program was 0–2 min: 98% A, 30% B; 2–5 min: 70% A, 30% B; 5–8 min: 20% A, 80% B; 8–8.1 min: 10% A, 90% B; and 8.1–10 min: 90% A, 10% B. The mass spectrometry scan range was selected as *m*/*z* 100–1500, the ESI source was set at a spray voltage of 3.5 kV, the sheath gas flow rate was 35 psi, the gas flow rate was 35 psi, the auxiliary gas flow rate was 10 L/min, the capillary temperature was 320 °C, and the S-lens radio frequency (RF) level was 60. The polarity was set to positive and negative, and the MS/MS secondary scan mode was data-dependent scans. KEGG enrichment analysis was performed on the significantly different metabolites.

#### 4.2.10. DNA Extraction and High-Throughput 16S rRNA Sequencing

Total DNA extraction was performed in strict accordance with the kit instructions of the FastDNA Spin Kit for Soil. DNA concentration and purity were measured using a NanoDrop 2000 ultra-micro spectrophotometer (Thermo Fisher Scientific), and DNA extraction quality was measured using 1% agarose gel electrophoresis. Polymerase chain reaction (PCR) was performed with 338F (5′-ACTCCTACGGGAGGCAGCAG-3′) and 806R (5′-GGACTACHVGGGTWTCTAAT-3′) primers for the V3-V4 hypervariable regions of the 16S ribosomal RNA (rRNA) gene, using an ABI GeneAmp 9700 PCR instrument (ABI, Foster City, CA, USA). The amplification procedures were 95 °C pre-denaturation for 3 min, 27 cycles (95 °C denaturation for 30 s, 55 °C annealing for 30 s, and 72 °C extension for 30 s), and 72 °C extension for 10 min. The amplification system was 20 μL, which included 4 μL FastPfu buffer (5×), 2.5 mM dNTPs, 0.8 μL primers (5 μM), 0.4 μL FastPfu polymerase, and 10 ng of DNA template. The PCR products were recovered using a 2% agarose gel, purified using the AxyPrep DNA Gel Extraction Kit, eluted with Tris-HCl, and detected by 2% agarose electrophoresis. Detection and quantification were performed using QuantiFluor-ST. Purified amplicons were combined in equimolar ratios and assessed for library quality. Paired-end sequencing (2 × 300 bp) was performed on the Illumina MiSeq sequencer (Illumina, San Diego, CA, USA).

#### 4.2.11. Statistical Analysis

GraphPad Prism 6.0.1 software (GraphPad Software, San Diego, USA) and IBM SPSS Statistics R26.0.0.0 software (International Business Machines Corporation, New York, NY, USA) were used to analyze and plot the data. Data are expressed as mean ± standard deviation (SD). The inter-group comparisons were based on one-way analysis of variance (ANOVA) to determine the statistically significant differences between groups. A value of *p* < 0.05 or *p* < 0.01 was considered statistically significant. For qualitative and quantitative analyses of metabolites, CD (Compound Discoverer 3.1) software was used for data preprocessing. The preprocessing was followed by assessments of molecular ion peaks and fragment ions for molecular formula prediction and comparison with mzCloud, mzVaulth, and MassList databases, removal of background ions with blank samples, and normalization of quantitative results. Finally, the data were identified, and quantitative results were obtained. Based on the R language MetaboAnalystR package, quality control of sample data, unsupervised dimensionality reduction analysis (PCA), OPLS-DA, univariate analysis, volcano plots, and screening of characteristic metabolites (biomarkers) were performed. The metabolites with *p* < 0.05 and VIP value > 1 were considered as differential metabolites. The 16S rRNA sequencing data were statistically analyzed using QIIME2 (2019.10) software. Differences in microbial community structure between samples were assessed based on the characteristic sequence level alpha diversity (α-diversity) index and beta diversity (β-diversity) index, followed by PCoA and NMDS plots for presentation. LEfSe and LDA were used to identify bacteria with differences in abundance between groups and samples. Taxa with LDA ≥ 2 were considered significantly different. Based on the relative abundance of major microbial species in the sample, the co-occurrence analysis was established by Spearman correlation coefficients to understand the associations among species. PICRUSt 1.1.4 software was applied to predict the possible functions of microbial groups and to perform differential functional analysis between groups.

## 5. Conclusions

**Figure 10:**
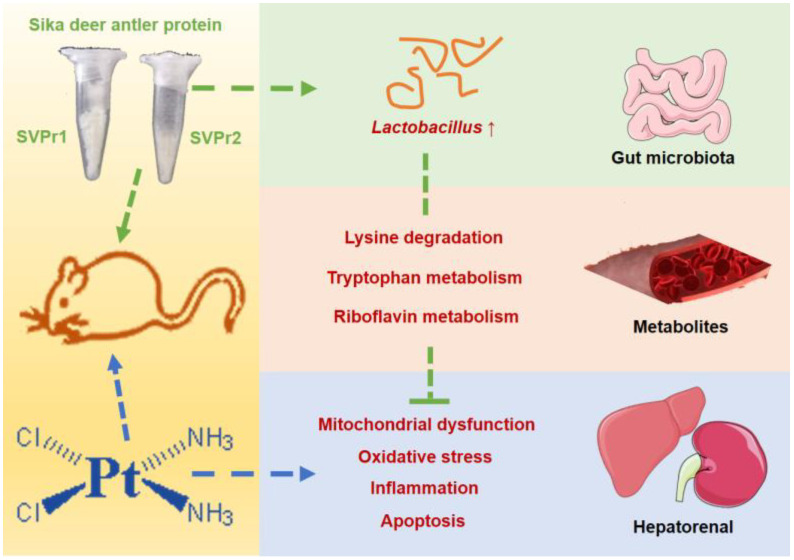
Underlying mechanisms of the protective effects of SVPr1 and SVPr2 on cisplatin-induced hepatorenal injury through the gut microbiota–metabolites axis.

In this study, we found that SVPr1 and SVPr2 had protective effects against cisplatin-induced hepatorenal injury. SVPr1 and SVPr2 ameliorated cisplatin-induced mitochondrial dysfunction, oxidative stress, inflammation, and apoptosis in the liver and kidney by modulating the levels of gut microbiota and associated metabolites. SVPr1 and SVPr2 mitigated cisplatin-induced impairment of mitochondrial function, probably by increasing the abundance of Lactobacillus and interfering with the lysine degradation pathway. Additionally, the attenuation of cisplatin-induced oxidative stress, inflammation, and apoptosis by SVPr1 and SVPr2 may be related to their modulation of the gut microbiota structure, particularly by increasing Lactobacillus abundance, inhibiting tryptophan metabolism and promoting riboflavin metabolic pathways (Figure 10). These findings may help in the development of novel therapeutic approaches to combat the adverse effects of chemotherapy drugs that involve targeting the composition and metabolites of the gut microbiota via dietary intervention.

## Figures and Tables

**Table 1:** The KEGG pathway enrichment result of differential metabolites between SVPr1 and CDDP groups.

No.	Metabolite	Formula	KEGG ID	*p*-Value	VIP	Trend Up/Down
1	Arachidonic acid	C_20_H_32_O_2_	C00219	0.006	2.206	down
2	Nervonic acid	C_24_H_46_O_2_	C08323	0.011	1.968	up
3	Taurochenodeoxycholic acid	C_26_H_45_NO_6_S	C05465	0.014	1.854	up
4	Acetyl-L-carnitine	C_9_H_17_NO_4_	C02571	0.014	1.845	down
5	L-Lysine	C_6_H_14_N_2_O_2_	C00047	0.019	1.731	down
6	Taurocholic acid	C_26_H_45_NO_7_S	C05122	0.020	1.701	down
7	L-2-Aminoadipic acid	C_6_H_11_NO_4_	C00956	0.026	1.591	down
8	4-Methylphenol	C_7_H_8_O	C01468	0.027	1.572	down
9	L-Saccharopine	C_11_H_2_0N_2_O_6_	C00449	0.029	1.536	down
10	Cytosine	C_4_H_5_N_3_O	C00380	0.036	1.447	up
11	Nicotinamide	C_6_H_6_N_2_O	C00153	0.036	1.440	up
12	L-Kynurenine	C_10_H_12_N_2_O_3_	C00328	0.037	1.434	down
13	D-Ribulose 5-phosphate	C_5_H_11_O_8_P	C00199	0.037	1.428	up
14	Taurine	C_2_H_7_NO_3_S	C00245	0.038	1.418	down
15	Pregnenolone	C_21_H_3_2O_3_	C01953	0.038	1.417	down
16	Cortolone	C_21_H_3_4O_5_	C05481	0.038	1.417	up
17	Glutathione	C_10_H_17_N_3_O_6_S	C00051	0.039	1.411	up
18	Xanthurenic Acid	C_10_H_7_NO_4_	C02470	0.040	1.394	up
19	3-Methylindole	C_9_H_9_N	C08313	0.042	1.376	down
20	Riboflavin	C_17_H_20_N_4_O_6_	C00255	0.043	1.369	down
21	O-Acetyl-L-carnitine	C_9_H_17_NO_4_	C02571	0.048	1.319	down

**Table 2:** The KEGG pathway enrichment result of differential metabolites between SVPr2 and CDDP groups.

No.	Metabolite	Formula	KEGG ID	*p*-Value	VIP	Trend Up/Down
1	L-Saccharopine	C_11_H_20_N_2_O_6_	C00449	0.005	2.271	down
2	L-Lysine	C_6_H_14_N_2_O_2_	C00047	0.006	2.241	down
3	Riboflavin	C_17_H_20_N_4_O_6_	C00255	0.006	2.213	down
4	Acetophenone	C_8_H_8_O	C07113	0.010	2.011	up
5	Taurocholic acid	C_26_H_45_NO_7_S	C05122	0.011	1.956	down
6	L-Kynurenine	C_10_H_12_N_2_O_3_	C00328	0.013	1.881	down
7	D-Ribulose 5-phosphate	C_5_H_11_O_8_P	C00199	0.018	1.739	up
8	3-Methylindole	C_9_H_9_N	C08313	0.020	1.694	down
9	Indole	C_8_H_7_N	C00463	0.021	1.677	down
10	L-Tryptophan	C_11_H_12_N_2_O_2_	C00078	0.021	1.670	up
11	Xanthurenic Acid	C_10_H_7_NO_4_	C02470	0.023	1.639	up
12	L-Adrenaline	C_9_H_13_NO_3_	C00788	0.023	1.632	down
13	Deoxycorticosterone	C_21_H_30_O_3_	C03205	0.026	1.591	up
14	3-Hydroxybutyric acid	C_4_H_8_O_3_	C01089	0.029	1.533	down
15	Arachidonic acid	C_20_H_32_O_2_	C00219	0.032	1.500	down
16	Cortisol	C_21_H_30_O_5_	C00735	0.034	1.472	down
17	Palmitoleic Acid	C_16_H_30_O_2_	C08362	0.036	1.447	down
18	Taurochenodeoxycholic acid	C_26_H_45_NO_6_S	C05465	0.038	1.418	down
19	Acetoacetate	C_4_H_6_O_3_	C00164	0.042	1.374	down
20	Aconitic acid	C_6_H_6_O_6_	C00417	0.047	1.327	down

## Data Availability

Data are contained within the article or Supplementary Materials.

## Supplementary Materials

The following supporting information can be downloaded at: https://www.mdpi.com/article/10.3390/molecules28186463/s1, Figure S1: Effects of SVPr1 and SVPr2 on the α and β diversity of gut microbiota; Figure S2: Representative total ion chromatograms (TICs) for different groups in positive and negative ion mode obtained from ultra-high performance liquid chromatography with quadrupole time-of-flight mass spectrometry (UHPLC-MS/MS) analysis; Figure S3: Principal component analysis (PCA) score plot for all samples in the ultra-high performance liquid chromatography–tandem mass spectrometry (UHPLC-MS/MS) system; Table S1: Results of liver index, left kidney index, right kidney index; Table S2: Identification results of differential metabolites in SVPr1 vs. CDDP groups; Table S3: Identification results of differential metabolites in SVPr2 vs. CDDP groups.

## Sample Availability

Samples are available from the authors upon request.

## References

[1] Ghosh S. (2019). Cisplatin: The First Metal Based Anticancer Drug. Bioorg Chem..

[2] Pabla N., Huang S., Mi Q.S., Daniel R., Dong Z. (2008). ATR-Chk2 Signaling in P53 Activation and DNA Damage Response during Cisplatin-Induced Apoptosis. J. Biol. Chem..

[3] Hu Y., Yang C., Amorim T., Maqbool M., Lin J., Li C., Fang C., Xue L., Kwart A., Fang H. (2021). Cisplatin-Mediated Upregulation of APE2 Binding to MYH9 Provokes Mitochondrial Fragmentation and Acute Kidney Injury. Cancer Res..

[4] Zhu X., Luo L., Xiong Y., Jiang N., Wang Y., Lv Y., Xie Y. (2022). VDAC1 Oligomerization May Enhance DDP-Induced Hepatocyte Apoptosis by Exacerbating Oxidative Stress and Mitochondrial DNA Damage. FEBS Open Bio.

[5] Tang C., Livingston M.J., Safirstein R., Dong Z. (2023). Cisplatin Nephrotoxicity: New Insights and Therapeutic Implications. Nat. Rev. Nephrol..

[6] Litterst C.L., Gram T.E., Dedrick R.L., Leroy A.F., Guarino A.M. (1976). Distribution and Disposition of Platinum Following Intravenous Administration of Cis-Diamminedichloroplatinum(II) (NSC 119875) to Dogs. Cancer Res..

[7] Kim J.S., Son J.Y., Kim K.S., Lim H.J., Ahn M.Y., Kwack S.J., Kim Y.M., Lee K.Y., Lee J., Lee B.M. (2018). Hepatic Damage Exacerbates Cisplatin-Induced Acute Kidney Injury in Sprague-Dawley Rats. J. Toxicol. Environ. Health. Part A.

[8] Galfetti E., Cerutti A., Ghielmini M., Zucca E., Wannesson L. (2020). Risk Factors for Renal Toxicity after Inpatient Cisplatin Administration. BMC Pharmacol. Toxicol..

[9] Wang J., Wei Y., Zhou Z., Yang J., Jia Y., Wu H., Dong H., Leng X. (2022). Deer Antler Extract Promotes Tibia Fracture Healing in Mice by Activating BMP-2/SMAD4 Signaling Pathway. J. Orthop. Surg. Res..

[10] Wu F., Li H., Jin L., Li X., Ma Y., You J., Li S., Xu Y. (2013). Deer Antler Base as a Traditional Chinese Medicine: A Review of Its Traditional Uses, Chemistry and Pharmacology. J. Ethnopharmacol..

[11] Chunhua M., Hongyan L. (2017). Protective Effect of Pilose Antler Peptide on Carbon Tetrachloride-Induced Hepatotoxicity in Mice. Int. J. Biol. Macromol..

[12] Ruan H., Luo J., Wang L., Wang J., Wang Z., Zhang J. (2019). Sika Deer Antler Protein against Acetaminophen-Induced Nephrotoxicity by Activating Nrf2 and Inhibition FoxO1 via PI3K/Akt Signaling. Int. J. Biol. Macromol..

[13] Sui Z., Zhang L., Huo Y., Zhang Y. (2014). Bioactive Components of Velvet Antlers and Their Pharmacological Properties. J. Pharm. Biomed. Anal..

[14] Yang H., Li W., Wang L., He X., Sun H., Zhang J. (2018). The Proteins from Sika Deer Antler as Potential Modulators on Cisplatin-Induced Cytotoxicity in Human Embryonic Kidney 293 Cells. Nat. Prod. Res..

[15] Tang Y., Fan M., Choi Y.J., Choi E.J., Moon S.H., Debnath T., Yu Y., Lee I.N., Kim E.K. (2018). Protective Effect of Sika Deer (Cervus Nippon) Velvet Antler Extract against Cisplatin-Induced Kidney and Liver Injury in a Prostate Cancer PC-3 Cell Xenograft Model. J. Chem..

[16] Qin J., Li Y., Cai Z., Li S., Zhu J., Zhang F., Liang S., Zhang W., Guan Y., Shen D. (2012). A Metagenome-Wide Association Study of Gut Microbiota in Type 2 Diabetes. Nature.

[17] Lun H., Yang W., Zhao S., Jiang M., Xu M., Liu F., Wang Y. (2019). Altered Gut Microbiota and Microbial Biomarkers Associated with Chronic Kidney Disease. MicrobiologyOpen.

[18] Vernocchi P., Del Chierico F., Putignani L. (2016). Gut Microbiota Profiling: Metabolomics Based Approach to Unravel Compounds Affecting Human Health. Front. Microbiol..

[19] Idborg H., Pawelzik S.C. (2022). Prostanoid Metabolites as Biomarkers in Human Disease. Metabolites.

[20] Zhang Z.W., Han P., Fu J., Yu H., Xu H., Hu J.C., Lu J.Y., Yang X.Y., Zhang H.J., Bu M.M. (2023). Gut Microbiota-Based Metabolites of Xiaoyao Pills (a Typical Traditional Chinese Medicine) Ameliorate Depression by Inhibiting Fatty Acid Amide Hydrolase Levels in Brain. J. Ethnopharmacol..

[21] Xia D., Lai X., Wu K., Zhou P., Li L., Guo Z., Xu S. (2019). Metabolomics Study of Fasudil on Cisplatin-Induced Kidney Injury. Biosci. Rep..

[22] Gong S., Feng Y., Zeng Y., Zhang H., Pan M., He F., Wu R., Chen J., Lu J., Zhang S. (2021). Gut Microbiota Accelerates Cisplatin-Induced Acute Liver Injury Associated with Robust Inflammation and Oxidative Stress in Mice. J. Transl. Med..

[23] Zhang Y., Li L., Qin S., Yuan J., Xie X., Wang F., Hu S., Yi Y., Chen M. (2022). C-Phycocyanin Alleviated Cisplatin-Induced Oxidative Stress and Inflammation via Gut Microbiota-Metabolites Axis in Mice. Front. Nutr..

[24] Turnbaugh P.J., Ley R.E., Mahowald M.A., Magrini V., Mardis E.R., Gordon J.I. (2006). An Obesity-Associated Gut Microbiome with Increased Capacity for Energy Harvest. Nature.

[25] Chen R., Wang J., Zhan R., Zhang L., Wang X. (2019). Fecal Metabonomics Combined with 16S RRNA Gene Sequencing to Analyze the Changes of Gut Microbiota in Rats with Kidney-Yang Deficiency Syndrome and the Intervention Effect of You-Gui Pill. J. Ethnopharmacol..

[26] Wu C.H., Ko J.L., Liao J.M., Huang S.S., Lin M.Y., Lee L.H., Chang L.Y., Ou C.C. (2019). D-Methionine Alleviates Cisplatin-Induced Mucositis by Restoring the Gut Microbiota Structure and Improving Intestinal Inflammation. Ther. Adv. Med. Oncol..

[27] Zong L., Li C., Zhong Y., Shi J., Yuan Z., Wang X. (2021). FTIR Microspectroscopic Investigation of Lactobacillus Paracasei Apoptosis Induced by Cisplatin. Spectrochim. Acta A Mol. Biomol. Spectrosc..

[28] Zick Y. (2001). Insulin Resistance: A Phosphorylation-Based Uncoupling of Insulin Signaling. Trends Cell Biol..

[29] Fox J.G., Rogers A.B., Whary M.T., Taylor N.S., Xu S., Feng Y., Keys S. (2004). Helicobacter Bilis-Associated Hepatitis in Outbred Mice. Comp. Med..

[30] Liu X.Z., Zhang Y.-M., Jia N.Y., Zhang H. (2020). Helicobacter Pylori Infection Is Associated with Elevated Galactose-Deficient IgA1 in IgA Nephropathy. Ren. Fail..

[31] Han S., Shang L., Lu Y., Wang Y. (2022). Gut Microbiome Characteristics in IgA Nephropathy: Qualitative and Quantitative Analysis from Observational Studies. Front. Cell Infect. Microbiol..

[32] Wang Y., Pan C.Q., Xing H. (2019). Advances in Gut Microbiota of Viral Hepatitis Cirrhosis. Biomed. Res. Int..

[33] Fang C., Zhou Q., Liu Q., Jia W., Xu Y. (2022). Crosstalk between Gut Microbiota and Host Lipid Metabolism in a Mouse Model of Alcoholic Liver Injury by Chronic Baijiu or Ethanol Feeding. Food Funct..

[34] Shao L., Chen Z., Soutto M., Zhu S., Lu H., Romero-Gallo J., Peek R., Zhang S., El-Rifai W. (2019). Helicobacter Pylori-Induced MiR-135b-5p Promotes Cisplatin Resistance in Gastric Cancer. FASEB J..

[35] Yang J., Ji G.E., Park M.S., Seong Y.J., Go Y.S., Lee H.Y., Fang Y., Kim M.G., Oh S.W., Cho W.Y. (2021). Probiotics Partially Attenuate the Severity of Acute Kidney Injury through an Immunomodulatory Effect. Kidney Res. Clin. Pract..

[36] Zhang T., Wang J., Yao Z., Ni L., Zhao Y., Wei S., Chen Z. (2022). Effect and Mechanism of Bifidobacterium Animalis B94 in the Prevention and Treatment of Liver Injury in Rats. Front. Cell Infect. Microbiol..

[37] Zhang Y., Qin S., Song Y., Yuan J., Hu S., Chen M., Li L. (2022). Alginate Oligosaccharide Alleviated Cisplatin-Induced Kidney Oxidative Stress via Lactobacillus Genus-FAHFAs-Nrf2 Axis in Mice. Front. Immunol..

[38] Ma K., Bai Y., Li J., Ren Z., Li J., Zhang J., Shan A. (2022). Lactobacillus Rhamnosus GG Ameliorates Deoxynivalenol-Induced Kidney Oxidative Damage and Mitochondrial Injury in Weaned Piglets. Food Funct..

[39] Lv L., Yao C., Yan R., Jiang H., Wang Q., Wang K., Ren S., Jiang S., Xia J., Li S. (2021). Lactobacillus Acidophilus LA14 Alleviates Liver Injury. mSystems.

[40] Mao R.W., He S.P., Lan J.G., Zhu W.Z. (2022). Honokiol Ameliorates Cisplatin-Induced Acute Kidney Injury via Inhibition of Mitochondrial Fission. Br. J. Pharmacol..

[41] Gaona-Gaona L., Molina-Jijón E., Tapia E., Zazueta C., Hernández-Pando R., Calderón-Oliver M., Zarco-Márquez G., Pinzón E., Pedraza-Chaverri J. (2011). Protective Effect of Sulforaphane Pretreatment against Cisplatin-Induced Liver and Mitochondrial Oxidant Damage in Rats. Toxicology.

[42] Leandro J., Houten S.M. (2020). The Lysine Degradation Pathway: Subcellular Compartmentalization and Enzyme Deficiencies. Mol. Genet. Metab..

[43] Zhou J., Wang X., Wang M., Chang Y., Zhang F., Ban Z., Tang R., Gan Q., Wu S., Guo Y. (2019). The Lysine Catabolite Saccharopine Impairs Development by Disrupting Mitochondrial Homeostasis. J. Cell Biol..

[44] Qu X., Gao H., Sun J., Tao L., Zhang Y., Zhai J., Song Y., Hu T., Li Z. (2020). Identification of Key Metabolites during Cisplatin-Induced Acute Kidney Injury Using an HPLC-TOF/MS-Based Non-Targeted Urine and Kidney Metabolomics Approach in Rats. Toxicology.

[45] Li Y., Liu X., Liu S., Lu J., Chen J., Xiong G., Yang S., Li S. (2020). Untargeted Metabolomics Reveals the Protective Effect of a Traditional Chinese Herbal Decoction on Cisplatin-Induced Acute Kidney Injury. Evid. Based Complement. Altern. Med. Ecam.

[46] Beier U.H., Hartung E.A., Concors S., Hernandez P.T., Wang Z., Perry C., Baur J.A., Denburg M.R., Hancock W.W., Gade T.P. (2020). Tissue Metabolic Profiling Shows That Saccharopine Accumulates during Renal Ischemic-Reperfusion Injury, While Kynurenine and Itaconate Accumulate in Renal Allograft Rejection. Metabolomics.

[47] Rinschen M.M., Palygin O., El-Meanawy A., Domingo-Almenara X., Palermo A., Dissanayake L.V., Golosova D., Schafroth M.A., Guijas C., Demir F. (2022). Accelerated Lysine Metabolism Conveys Kidney Protection in Salt-Sensitive Hypertension. Nat. Commun..

[48] Padilla P., Andrade M.J., Peña F.J., Rodríguez A., Estévez M. (2022). An in Vitro Assay of the Effect of Lysine Oxidation End-Product, α-Aminoadipic Acid, on the Redox Status and Gene Expression in Probiotic Lactobacillus Reuteri PL503. Amino Acids.

[49] Jiang L., Wang J., Xu L., Cai J., Zhao S., Ma A. (2022). Lactobacillus Casei Modulates Inflammatory Cytokines and Metabolites during Tuberculosis Treatment: A Post Hoc Randomized Controlled Trial. Asia Pac. J. Clin. Nutr..

[50] Zhang P., Chen J.Q., Huang W.Q., Li W., Huang Y., Zhang Z.J., Xu F.G. (2017). Renal Medulla Is More Sensitive to Cisplatin than Cortex Revealed by Untargeted Mass Spectrometry-Based Metabolomics in Rats. Sci. Rep..

[51] Tan B., Chen J., Qin S., Liao C., Zhang Y., Wang D., Li S., Zhang Z., Zhang P., Xu F. (2021). Tryptophan Pathway-Targeted Metabolomics Study on the Mechanism and Intervention of Cisplatin-Induced Acute Kidney Injury in Rats. Chem. Res. Toxicol..

[52] Song Y., Hu T., Gao H., Zhai J., Gong J., Zhang Y., Tao L., Sun J., Li Z., Qu X. (2021). Altered Metabolic Profiles and Biomarkers Associated with Astragaloside IV-Mediated Protection against Cisplatin-Induced Acute Kidney Injury in Rats: An HPLC-TOF/MS-Based Untargeted Metabolomics Study. Biochem. Pharmacol..

[53] Lamas B., Richard M.L., Leducq V., Pham H.P., Michel M.L., Da Costa G., Bridonneau C., Jegou S., Hoffmann T.W., Natividad J.M. (2016). CARD9 Impacts Colitis by Altering Gut Microbiota Metabolism of Tryptophan into Aryl Hydrocarbon Receptor Ligands. Nat. Med..

[54] Cervantes Barragan L., Chai J.N., Tianero M.D., Di Luccia B., Ahern P.P., Merriman J., Cortez V.S., Caparon M.G., Donia M.S., Gilfillan S. (2017). Lactobacillus Reuteri Induces Gut Intraepithelial CD4+CD8αα+ T Cells. Science.

[55] Wrzosek L., Ciocan D., Hugot C., Spatz M., Dupeux M., Houron C., Lievin-Le Moal V., Puchois V., Ferrere G., Trainel N. (2021). Microbiota Tryptophan Metabolism Induces Aryl Hydrocarbon Receptor Activation and Improves Alcohol-Induced Liver Injury. Gut.

[56] Hj P. (2003). Riboflavin (Vitamin B-2) and Health. Am. J. Clin. Nutr..

[57] Hassan I., Chibber S., Naseem I. (2010). Ameliorative Effect of Riboflavin on the Cisplatin Induced Nephrotoxicity and Hepatotoxicity under Photoillumination. Food Chem. Toxicol..

[58] Liu Y., Deng Y., Wang F., Liu X., Wang J., Xiao J., Zhang C., Zhang Q. (2022). A New Mechanism for Ginsenoside Rb1 to Promote GLUCOSE Uptake, Regulating Riboflavin Metabolism and Redox Homeostasis. Metabolites.

