# Supplementary material for: Gut Microbiota Combined with Metabolomics Reveal the Mechanisms of Sika Deer Antler Protein on Cisplatin-Induced Hepatorenal Injury in Mice
Source: Molecules. 2023 Sep 6;28(18):6463. doi: 10.3390/molecules28186463 (PMC10537820; doi:10.3390/molecules28186463)
Supplement: Supplementary file 1 [file molecules-28-06463-s001.zip › molecules-2535948-supplementary.pdf]

Table S1. Results of liver index, left kidney index, right kidney index.

Items	CON	CDDP	SVPr1	SVPr2
Liver index (%)	3.80±0.39**	6.16±0.44##	4.27±0.42**	4.23±0.49**
Left kidney index (%)	0.57±0.07	0.61±0.09	0.58±0.11	0.60±0.08
Right kidney index (%)	0.59±0.05	0.63±0.04	0.59±0.12	0.58±0.03

** $p < 0.01$ vs. CDDP group; ## $p < 0.01$ vs. CON group.

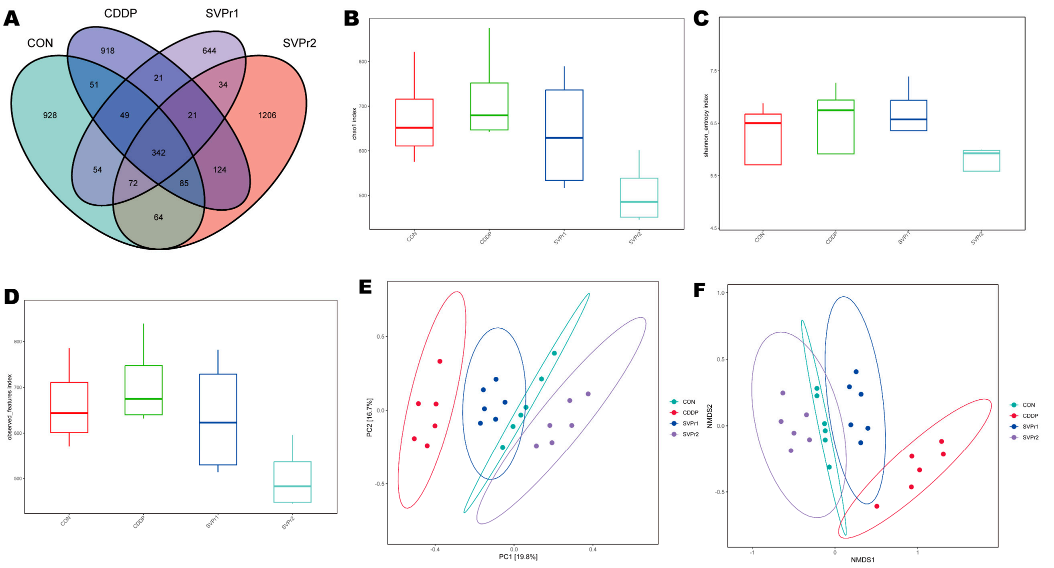

Figure S1. Effects of SVPr1 and SVPr2 on the α and β diversity of gut microbiota. (A) Venn diagram of the numbers of operational taxonomic units (OTUs) in the CON, CDDP, and SVPr1 and SVPr2 treatment groups. (B–D) α diversity assessment with Chao 1 (B), Shannon (C), and observed species (D) indices for each group. (E–F) β diversity assessment with principal coordinate analysis (PCoA) (E) and non-metric multidimensional scaling (NMDS) (F) analysis for each group. In the score plots, each symbol represents a sample (n=6 per group), and the samples are color-coded according to their group information. The axes represent the two dimensions that explain the greatest ratios of variance in the communities.

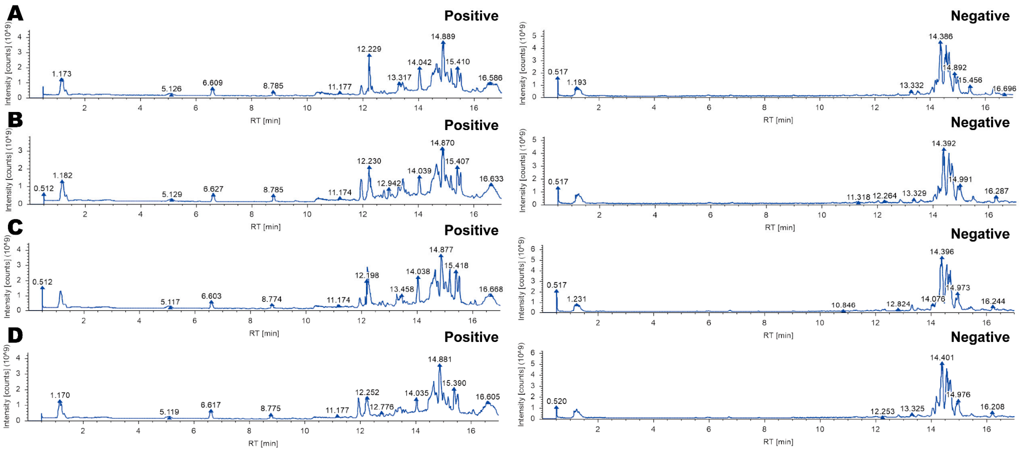

Figure S2. Representative total ion chromatograms (TICs) for different groups in positive and negative ion mode obtained from ultra-high performance liquid chromatography with quadrupole time-of-flight mass spectrometry (UHPLC-MS/MS) analysis. (A) Control group. (B) CDDP group. (C) SVPr1+CDDP group. (D) SVPr2+CDDP group. The left sides of the TICs indicate the positive modes, and the right sides indicate the negative modes.

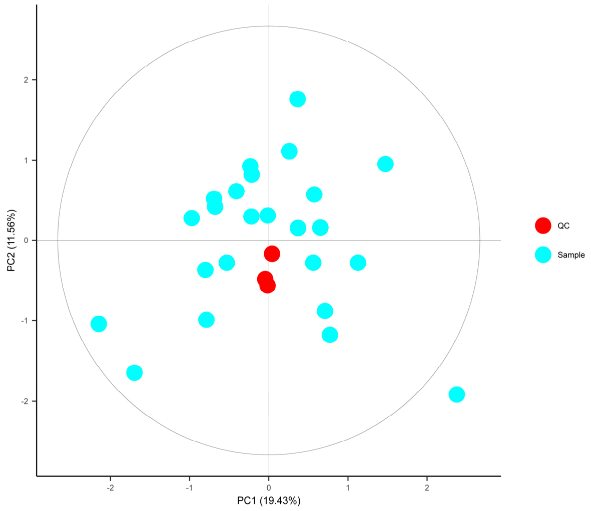

Figure S3. Principal component analysis (PCA) score plot for all samples in the ultra-high performance liquid chromatography–tandem mass spectrometry (UHPLC-MS/MS) system. QC, quality contro

Table S2. Identification results of differential metabolites in .SVPr1 vs. CDDP groups.

No.	Mode	Metabolite	Formula	Molecular Weight	tR/min	m/z	*p*-Value	VIP	Trend up/down
1	pos	Indole-3-lactic acid	$C_{11}H_{11}NO_3$	205.07381	9.092	206.08104	0.001	2.955	down
2	neg	Arachidonic acid	$C_{20}H_{32}O_2$	304.24	14.349	303.2326	0.006	2.206	down
3	pos	Indole-3-acrylic acid	$C_{11}H_9NO_2$	187.06325	9.146	188.07045	0.007	2.145	down
4	pos	PC (22:4e/18:3)	$C_{48}H_{84}NO_7P$	817.59752	16.014	818.60498	0.007	2.142	up
5	neg	Lysopc 17:0	$C_{25}H_{52}NO_7P$	509.34804	15.311	508.34076	0.008	2.078	down
6	neg	Nervonic acid	$C_{24}H_{46}O_2$	366.34989	15.855	365.34259	0.011	1.968	up
7	pos	PE (17:1/17:1)	$C_{39}H_{74}NO_8P$	715.51495	16.503	716.52252	0.014	1.862	up
8	neg	Taurochenodeoxycholic acid	$C_{26}H_{45}NO_6S$	499.29667	12.804	498.28949	0.014	1.854	up
9	pos	Acetyl-L-carnitine	$C_9H_{17}NO_4$	203.11568	9.472	204.12289	0.014	1.845	down
10	neg	3-Indoxyl sulphate	$C_8H_7NO_4S$	213.00964	6.019	212.0024	0.015	1.832	down
11	pos	Propionyl-L-carnitine	$C_{10}H_{19}NO_4$	217.13136	8.055	218.13858	0.015	1.830	up
12	pos	PC (19:2/18:3)	$C_{45}H_{80}NO_8P$	793.5606	16.578	794.56927	0.015	1.817	up
13	neg	(+/-)19(20)-DiHDPA	$C_{22}H_{34}O_4$	362.24599	12.725	361.23892	0.016	1.786	down
14	neg	LPI 20:4	$C_{29}H_{49}O_{12}P$	620.2982	13.907	619.29089	0.017	1.757	down
15	pos	D-threo-Isocitric acid	$C_6H_8O_7$	192.02687	2.041	215.01613	0.018	1.754	down
16	pos	5-oxoproline	$C_5H_7NO_3$	129.04259	12.14	130.04985	0.018	1.751	down
17	neg	SM (d14:2/28:0)	$C_{47}H_{93}N_2O_6P$	872.70251	14.495	871.69537	0.018	1.733	up
18	pos	L-lysine	$C_6H_{14}N_2O_2$	146.10542	1.233	147.11269	0.019	1.731	down
19	neg	Taurocholic acid	$C_{26}H_{45}NO_7S$	515.29404	12.119	1029.58081	0.020	1.701	down
20	neg	LPC 18:0	$C_{26}H_{54}NO_7P$	583.38563	15.313	582.3783	0.024	1.624	down
21	pos	L-2-Aminoadipic acid	$C_6H_{11}NO_4$	161.06861	2.029	162.07597	0.026	1.591	down
22	pos	3,5-Diiodo-L-thyronine	$C^{15}H_{13}I_2NO_4$	524.89319	1.226	1072.77527	0.026	1.581	up

Table S2. Cont.

No.	Mode	Metabolite	Formula	Molecular Weight	tR/min	m/z	*p*-Value	VIP	Trend up/down
23	pos	3-[2-(3-Hydroxyphenyl)ethyl]-5-methoxyphenol	$C_{15}H_{16}O_3$	244.10972	10.521	245.11707	0.027	1.573	up
24	neg	4-Methylphenol	C_7H_8O	108.05767	7.803	107.05051	0.027	1.572	down
25	pos	L-Saccharopine	$C_{11}H_{20}N_2O_6$	276.13335	12.431	277.14041	0.029	1.536	down
26	pos	SM (d21:2/21:0)	$C_{47}H_{93}N_2O_6P$	812.67729	13.534	813.68457	0.032	1.497	down
27	neg	OxPC (18:0-20:3+1O(1Cyc))	$C_{46}H_{84}NO_9P$	885.61392	14.853	884.60614	0.032	1.491	down
28	neg	3-(1-cyano-1,2-dihydroisoquinolin-2-yl)-3-oxopropyl propionate	$C_{16}H_{16}N_2O_3$	284.11761	12.397	283.11026	0.033	1.479	down
29	pos	D-Erythro-sphingosine 1-phosphate	$C_{18}H_{38}NO_5P$	379.24815	17.067	380.2554	0.034	1.466	down
30	pos	Cytosine	$C_4H_5N_3O$	111.04347	1.592	112.05074	0.036	1.447	up
31	pos	Nicotinamide	$C_6H_6N_2O$	122.04809	1.863	123.05537	0.036	1.440	up
32	pos	L-Kynurenine	$C_{10}H_{12}N_2O_3$	208.08466	4.248	209.09193	0.037	1.434	down
33	pos	PC (19:1/18:2)	$C_{45}H_{84}NO_8P$	797.59147	16.36	798.59802	0.037	1.431	up
34	neg	D-Ribulose 5-phosphate	$C_5H_{11}O_8P$	230.01907	1.155	229.0118	0.037	1.428	up
35	neg	Taurine	$C_2H_7NO_3S$	125.01476	1.211	124.0075	0.038	1.418	down
36	neg	Pregnenolone	$C_{21}H_{32}O_3$	332.23524	13.542	331.22784	0.038	1.417	down
37	pos	Cortolone	$C_{21}H_{34}O_5$	348.22951	11.521	349.23676	0.038	1.417	up
38	pos	Glutathione	$C_{10}H_{17}N_3O_6S$	307.08339	1.807	308.09067	0.039	1.411	up
39	pos	PC (18:2/22:2)	$C_{48}H_{88}NO_8P$	837.61748	16.742	838.62476	0.040	1.403	up
40	pos	2-Linoleoyl glycerol	$C_{21}H_{38}O_4$	336.26388	15.377	337.271	0.040	1.399	up
41	pos	Xanthurenic Acid	$C_{10}H_7NO_4$	205.03749	7.043	206.04474	0.040	1.394	up
42	pos	3-Methylindole	C_9H9N	131.07352	9.141	132.08075	0.042	1.376	down
43	pos	Riboflavin	$C_{17}H_{20}N_4O_6$	376.13779	8.228	377.14496	0.043	1.369	down

Table S2. Cont.

No.	Mode	Metabolite	Formula	Molecular Weight	tR/min	m/z	*p*-Value	VIP	Trend up/down
44	pos	PE (18:1/20:5)	$C_{43}H_{74}NO_8P$	763.51472	16.151	764.52173	0.044	1.360	up
45	pos	PC (20:3e/22:4)	$C_{50}H_{88}NO_7P$	845.62692	15.297	846.63416	0.048	1.321	up
46	pos	O-Acetyl-L-carnitine	$C_9H_{17}NO_4$	203.11568	9.472	204.12289	0.048	1.319	down
47	neg	SM (d14:2/22:0)	$C_{41}H_{81}N_2O_6P$	788.60786	13.957	787.60059	0.049	1.314	down
48	pos	3-hydroxy-3-methylpentanedioic acid	$C_6H_{10}O_5$	184.03461	1.204	185.0419	0.049279	1.3073	down

Table S3. Identification results of differential metabolites in .SVPr2 vs. CDDP groups.

No.	Mode	Metabolite	Formula	Molecular Weight	tR/min	m/z	*p*-Value	VIP	Trend up/down
1	pos	L-Norvaline	$C_5H_{11}NO_2$	117.07904	1.674	118.08635	0.001	3.025	down
2	neg	LPC 22:1	$C_{30}H_{60}NO_7P$	637.4339	16.128	636.42682	0.002	2.767	down
3	neg	LPC 18:0	$C_{26}H_{54}NO_7P$	583.38563	15.313	582.3783	0.004	2.383	down
4	pos	L-Saccharopine	$C_{11}H_{20}N_2O_6$	276.13335	12.431	277.14041	0.005	2.271	down
5	neg	Lysopc 17:0	$C_{25}H_{52}NO_7P$	509.34804	15.311	508.34076	0.005	2.271	down
6	pos	L-lysine	$C_6H_{14}N_2O_2$	146.10542	1.233	147.11269	0.006	2.241	down
7	pos	Riboflavin	$C_{17}H_{20}N_4O_6$	376.13779	8.228	377.14496	0.006	2.213	down
8	neg	LPI 22:6	$C_{31}H_{49}O_{12}P$	644.29797	13.888	643.29059	0.007	2.170	down
9	pos	ACar 18:0	$C_{25}H_{50}NO_4$	427.36558	13.962	428.37283	0.008	2.114	down
10	pos	Acetophenone	C_8H_8O	120.05756	12.82	121.06478	0.010	2.011	up
11	neg	Taurocholic acid	$C_{26}H_{45}NO_7S$	515.29404	12.119	1029.58081	0.011	1.956	down
12	pos	1-Methylguanine	$C_6H_7N_5O$	165.06489	1.876	166.07222	0.012	1.919	down

Table S3. Cont.

No.	Mode	Metabolite	Formula	Molecular Weight	tR/min	m/z	*p*-Value	VIP	Trend up/down
13	neg	3-Indoxyl sulphate	$C_8H_7NO_4S$	213.00964	6.019	212.0024	0.012	1.917	down
14	pos	L-Kynurenine	$C_{10}H_{12}N_2O_3$	208.08466	4.248	209.09193	0.013	1.881	down
15	neg	D-Ribulose 5-phosphate	$C_5H_{11}O_8P$	230.01907	1.155	229.0118	0.018	1.739	up
16	pos	3-hydroxy-3-methylpentanedioic acid	$C_6H_{10}O_5$	184.03461	1.204	185.0419	0.019	1.722	down
17	pos	PC (18:2/18:2)	$C_{44}H_{80}NO_8P$	781.56165	16.909	782.56915	0.020	1.700	up
18	pos	Indole-3-lactic acid	$C_{11}H_{11}NO_3$	205.07381	9.092	206.08104	0.020	1.699	down
19	pos	3-Methylindole	C_9H_9N	131.07352	9.141	132.08075	0.020	1.694	down
20	pos	ethyl 1-(2-naphthylsulfonyl)-4-piperidinecarboxylate	$C_{18}H_{21}NO_4S$	347.1138	11.987	348.12152	0.021	1.686	up
21	pos	Indole	C_8H_7N	117.058	6.661	118.06531	0.021	1.677	down
22	pos	PC (18:2/18:3)	$C_{44}H_{78}NO_8P$	779.5457	16.133	780.55298	0.021	1.672	up
23	pos	L-Tryptophan	$C_{11}H_{12}N_2O_2$	204.0897	6.661	205.09697	0.021	1.670	up
24	pos	6-Methylquinoline	$C_{10}H_9N$	143.07344	6.661	144.08069	0.022	1.662	down
25	pos	Xanthurenic Acid	$C_{10}H_7NO_4$	205.03749	7.043	206.04474	0.023	1.639	up
26	neg	1,2,3-cyclopropanetricarboxylic acid	$C_6H_6O_6$	174.01645	1.128	173.00917	0.023	1.636	down
27	pos	L-Adrenaline	$C_9H_{13}NO_3$	183.08951	6.167	184.0968	0.023	1.632	down
28	pos	5-Hydroxytryptophan	$C_{11}H_{12}N_2O_3$	220.08466	6.664	221.09181	0.023	1.630	down
29	pos	Deoxycorticosterone	$C_{21}H_{30}O_3$	330.21882	13.524	331.22623	0.026	1.591	up
30	neg	3-Hydroxybutyric acid	$C_4H_8O_3$	104.04754	1.307	103.04027	0.029	1.533	down
31	pos	DL-Dipalmitoylphosphatidylcholine	$C_{40}H_{80}NO_8P$	733.56263	16.31	734.56952	0.031	1.508	up

Table S3. Cont.

No.	Mode	Metabolite	Formula	Molecular Weight	tR/min	m/z	*p*-Value	VIP	Trend up/down
32	neg	Arachidonic acid	$C_{20}H_{32}O_2$	304.24	14.349	303.2326	0.032	1.500	down
33	neg	PG (11:0/18:2)	$C_{35}H_{65}O_{10}P$	676.4237	14.409	675.4173	0.032	1.494	up
34	pos	Cortisol	$C_{21}H_{30}O_5$	362.20875	11.606	363.21588	0.034	1.472	down
35	pos	Palmitoleic Acid	$C_{16}H_{30}O_2$	276.20587	1.207	277.21298	0.036	1.447	down
36	neg	FAHFA (18:2/18:1)	$C_{36}H_{64}O_4$	560.48132	14.424	559.47406	0.036	1.444	down
37	neg	Taurochenodeoxycholic acid	$C_{26}H_{45}NO_6S$	499.29667	12.804	498.28949	0.038	1.418	down
38	pos	PC (20:3/20:3)	$C_{48}H_{84}NO_8P$	833.59305	16.219	834.60028	0.039	1.408	up
39	pos	PC (17:0/18:3)	$C_{43}H_{80}NO_8P$	769.5615	16.838	770.56952	0.041	1.385	up
40	pos	D-threo-Isocitric acid	$C_6H_8O_7$	192.02687	2.041	215.01613	0.042	1.377	down
41	neg	Acetoacetate	$C_4H_6O_3$	102.03171	1.11	101.02437	0.042	1.374	down
42	pos	Propionylcarnitine	$C_{10}H_{19}NO_4$	217.13123	2.67	218.13855	0.046	1.335	down
43	pos	PC (20:3/20:4)	$C_{48}H_{82}NO_8P$	831.57682	16.859	832.58441	0.046	1.333	up
44	neg	Erythronolactone	$C_4H_6O_4$	118.02671	1.147	117.0193	0.047	1.331	down
45	neg	Aconitic acid	$C_6H_6O_6$	192.02696	1.172	191.0197	0.047	1.327	down
46	pos	Linolelaidic Acid (C18:2N6T)	$C_{18}H_{32}O_2$	280.23977	14.213	281.24698	0.048	1.317	down
47	neg	LysoPE 18:0	$C_{23}H_{48}NO_7P$	481.3167	14.923	480.30914	0.048	1.315	down
48	pos	8-Hydroxyquinoline	C_9H_7NO	145.05266	8.48	146.05994	0.049	1.314	down
49	pos	N-Phenylacetylglutamine	$C_{13}H_{16}N_2O_4$	264.11065	7.754	265.11783	0.049	1.3131	down
